# Guidelines, Consensus Statements, and Standards for the Use of Artificial Intelligence in Medicine: Systematic Review

**DOI:** 10.2196/46089

**Published:** 2023-11-22

**Authors:** Ying Wang, Nian Li, Lingmin Chen, Miaomiao Wu, Sha Meng, Zelei Dai, Yonggang Zhang, Mike Clarke

**Affiliations:** 1 Department of Medical Administration West China Hospital Sichuan University Chengdu China; 2 Department of Anesthesiology, National Clinical Research Center for Geriatrics West China Hospital Sichuan University Chengdu China; 3 Department of General Practice, National Clinical Research Center for Geriatrics, International Medical Center West China Hospital Sichuan University Chengdu China; 4 Department of Operation Management West China Hospital Sichuan University Chengdu China; 5 Department of Radiation Oncology, Cancer Center and State Key Laboratory of Biotherapy West China Hospital Sichuan University Chengdu China; 6 Department of Periodical Press, National Clinical Research Center for Geriatrics, Chinese Evidence-based Medicine Center, Nursing Key Laboratory of Sichuan Province West China Hospital Sichuan University Chengdu China; 7 Northern Ireland Methodology Hub Queen's University Belfast Belfast United Kingdom

**Keywords:** artificial intelligence, clinical practice, guidelines, consensus statements, standards, systematic review

## Abstract

**Background:**

The application of artificial intelligence (AI) in the delivery of health care is a promising area, and guidelines, consensus statements, and standards on AI regarding various topics have been developed.

**Objective:**

We performed this study to assess the quality of guidelines, consensus statements, and standards in the field of AI for medicine and to provide a foundation for recommendations about the future development of AI guidelines.

**Methods:**

We searched 7 electronic databases from database establishment to April 6, 2022, and screened articles involving AI guidelines, consensus statements, and standards for eligibility. The AGREE II (Appraisal of Guidelines for Research & Evaluation II) and RIGHT (Reporting Items for Practice Guidelines in Healthcare) tools were used to assess the methodological and reporting quality of the included articles.

**Results:**

This systematic review included 19 guideline articles, 14 consensus statement articles, and 3 standard articles published between 2019 and 2022. Their content involved disease screening, diagnosis, and treatment; AI intervention trial reporting; AI imaging development and collaboration; AI data application; and AI ethics governance and applications. Our quality assessment revealed that the average overall AGREE II score was 4.0 (range 2.2-5.5; 7-point Likert scale) and the mean overall reporting rate of the RIGHT tool was 49.4% (range 25.7%-77.1%).

**Conclusions:**

The results indicated important differences in the quality of different AI guidelines, consensus statements, and standards. We made recommendations for improving their methodological and reporting quality.

**Trial Registration:**

PROSPERO International Prospective Register of Systematic Reviews (CRD42022321360); https://www.crd.york.ac.uk/prospero/display_record.php?RecordID=321360

## Introduction

The application of artificial intelligence (AI) has become increasingly common in the medical field. AI has been widely used in medical imaging [1], disease screening [2], the prediction and evaluation of treatments [3], the development of disease models from patient trajectories [4], and other areas. AI is expected to make medicine-related practice, research, and applications more accurate [5]. For example, a systematic review found 78 studies on the use of AI applications for COVID-19 up to September 19, 2020 [6]. Undoubtedly, this is a promising area, but as an emerging technology with a lot of gaps and grey zones to fill in, AI has prompted concerns about safety, accuracy, and applicability [7-10]. To promote the standardized application of AI in medicine, AI guidelines, consensus statements, and standards on various topics have been developed, and they contain recommendations aimed at improving patient care and use evidence from systematic reviews and assessments of potential benefits and harms [11]. Guidelines that are clear, precise, and transparent can assist health care practitioners, administrators, program managers, and the general public in understanding and implementing recommendations that will support and improve applications in medicine [11,12]. With the likely further clinical translation of AI systems, it will become increasingly important for AI guidelines to be both followed and regularly updated [13]. Therefore, high-quality guidelines on AI medicine would help professionals to improve decision-making and incorporate the best evidence into AI systems. Up to now, although many AI guidelines, consensus statements, and standards have been published in the medical field, quality issues resulting from their applications still exist [14,15], and low-quality guidelines might lead to widespread use of poor treatments and wasteful practices, which can ultimately harm patients [16].

To assess the scale of these issues, the current content and quality of AI guidelines in the medical field need to be evaluated, whether in medical research, medical practice, or other applications. We are not aware of any existing systematic review on this topic. In addition, the AGREE II (Appraisal of Guidelines for Research & Evaluation II) instrument is the most commonly used guideline appraisal tool. According to the latest description on the official website of the RIGHT (Reporting Items for Practice Guidelines in Healthcare) checklist, it can be used to evaluate the reporting quality of guidelines [17].

Therefore, in this systematic review, we used the RIGHT [17] and AGREE II [18] tools to assess the quality of guidelines in the field of AI for medicine and to provide a foundation for recommendations about the future development of AI guidelines.

## Methods

### Search Strategies

The authors agreed on the search strategies that were used to search the following databases from database establishment to April 6, 2022: PubMed, Web of Science, Embase, CNKI (Chinese National Knowledge Infrastructure), VIP, WanFang Data, and Sinomed. A manual search of unpublished literature (including conference proceedings, theses, dissertations, and grey literature) was also conducted. Only Chinese and English articles were included. The search strategies are presented in Multimedia Appendix 1.

### Inclusion and Exclusion Criteria

We included published and grey literature reports of guidelines, consensus statements, and standards according to the following criteria: (1) The purpose of the report should be to provide recommendations or statements on the application of AI in any medical field, with no limitations on the subject; and (2) Considering the title, abstract, and full text, the report should involve guidelines, consensus statements, or standards. We excluded the following types of papers: (1) Duplicate literature (only 1 article of the same report published in a different journal was retained); (2) Systematic reviews, meta-analyses, narrative reviews, literature reviews, and scoping reviews; (3) Reports related to only the guideline development process; (4) Conference abstracts; (5) Letters; and (6) Protocols. If more than one version of the report on guidelines, consensus statements, or standards was identified, we include the most recent version only.

### Study Selection

The results of the search were entered into the reference management program NoteExpress 3.4.0.8878 (Beijing Aegean Sea Lezhi Technology Co, Ltd). After removing duplicates, 2 authors examined the titles and abstracts of all included references, and deleted the literature that did not meet the theme. Then, the full text was retrieved for further screening, and a decision was made on eligibility for review. We removed research that lacked implementation suggestions or only included a summary statement or introduction. Disagreements were resolved by discussion or consultation with a third author.

### Data Extraction

For each study, the data extraction was conducted by 2 authors and then validated by another author. The extracted information included title, author, publication year, region, country, publishing organization, journal, number of authors, number of pages, number of references, registration or no registration, methods used to form recommendations, research subjects, use of AI, etc. In this study, strict and detailed data collection methods were formulated, and data extraction personnel were trained intensively. Disagreements were resolved by discussion.

### Evaluation of Quality

Using the AGREE II tool, 4 authors independently assessed the methodological quality of each included guideline and consensus statement [18]. The appraisal included an examination of technical and supporting materials, including (1) Scope and purpose; (2) Stakeholder involvement; (3) Rigor of development; (4) Clarity of presentation; (5) Applicability; and (6) Editorial independence, using the 23 components in the 6 domains of the AGREE II tool. Each item was graded on a 7-point scale, with 1 indicating significant disagreement and 7 indicating strong agreement. Each domain score was generated in Excel (Microsoft Corp) from its item scores by the AGREE II tool, with a minimum domain score of 0% and a maximum domain score of 100%. We analyzed the scores of items inside each domain and produced a scaled domain score for each domain for each guideline according to the AGREE II manual as follows: (obtained score – minimum score) / (maximum score – minimum score) × 100%. The overall mean score was derived using the average score of the 6 domains. We used an intraclass correlation coefficient (ICC) consistency analysis to analyze the κ value for the 4 evaluations. A κ value of >0.7 suggested strong consistency, while a value of 0.4 suggested low consistency.

Four researchers also used the RIGHT checklist to assess the quality of reporting. The RIGHT checklist has 7 domains, with 22 items and 35 subitems: basic information (items 1-4), background (items 5-9), evidence (items 10-12), recommendations (items 13-15), review and quality assurance (items 16 and 17), funding and declaration and management of interests (items 18 and 19), and other information (items 20-22). We provided a binary score of “Yes” (compliant) or “No” (not compliant) for each item. We analyzed the reporting rate for the results of the RIGHT assessment as number of compliant subitems/total sub items × 100%. A higher value indicated a higher reporting quality. We reached a single decision through discussion if there were contradictions in the evaluation results of the 4 researchers.

### Systematic Review and Statistical Analysis

This study followed the PRISMA (Preferred Reporting Items for Systematic Reviews and Meta-Analyses) checklist (Multimedia Appendix 2). The systematic review was performed to analyze the quality of the guidelines, consensus statements, and standards, and tabulate the study intervention characteristics. Moreover, a content analysis was performed to compare and contrast the recommendations. Guidelines, consensus statements, and standards were classified and analyzed, and different applications and development trends were classified and analyzed. SPSS 21.0 software (IBM Corp) was used for data analysis. The ICCs between the 4 reviewers were high (>0.7). 

## Results

### Results of the Literature Search

**Figure 1:**
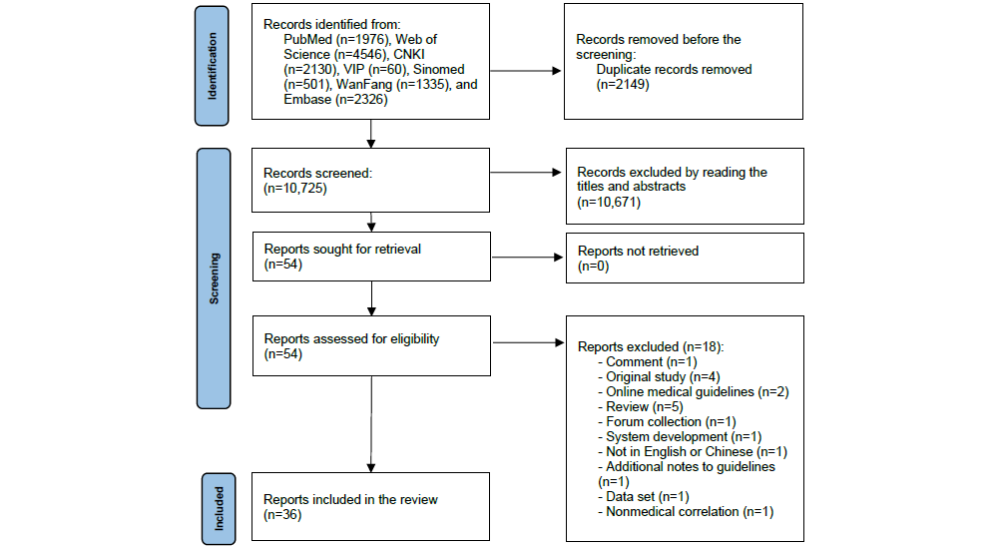
Flow diagram of the search and selection of articles involving guidelines, consensus statements, and standards. CNKI: China National Knowledge Infrastructure.

The searches retrieved 12,874 articles. A total of 2149 articles were excluded owing to duplication and 10,671 were excluded after title or abstract screening, leaving 54 full-text articles for further evaluation. The eligibility of the 54 articles was determined, and 18 were excluded for the following reasons: comment (n=1), original study (n=4), online medical guidelines (n=2), review (n=5), forum collection (n=1), system development (n=1), not in English or Chinese (n=1), additional notes to guidelines (n=1), data set (n=1), and nonmedical correlation (n=1). The remaining 36 articles were included (19 articles involved guidelines, 14 involved consensus statements, and 3 involved standards). The screening process is summarized in the PRISMA flowchart (Figure 1).

### Characteristics of the Included Articles Involving Guidelines

Considering the full text, we defined the distinction of guidelines, consensus statements, and standards. The included articles involving guidelines (n=19), consensus statements (n=14), and standards (n=3) were published from 2019 to 2022.

Of the 36 articles, 16 were from European and American countries, and 20 were from China and Australia (Multimedia Appendix 3). Nine articles were related to disease screening, diagnosis, or treatment, including retinal screening (n=2), breast disease screening (n=1), esophageal cancer diagnosis and treatment (n=1), colorectal tumor (n=2), glaucoma auxiliary screening (n=1), and pulmonary nodules (n=2). Three articles involved methodological guidelines for the reporting of AI interventions. Five articles aimed to guide clinical practitioners to apply AI imaging and promote radiology development and cooperation. Fourteen articles involved AI data acquisition, labeling and calculation of digestive endoscopy, central nervous system tumor, local lesion of the liver, colorectal cancer, chest radiography, eye disease imaging, body composition assessment, personalized medicine, biomedical data set construction, population-based health indicators, AI electrocardiography (ECG), pneumoconiosis, and pulmonary nodules on chest computed tomography (CT). Five articles were related to AI ethics and governance.

The included guidelines, consensus statements, and standards were related to general surgery, ophthalmology, radiology, dermatology, oncology, etc, involving diabetic retinopathy, tumors, and other related diseases (Table 1).

**Table 1:** Summary of included guidelines and consensus statements.

Classification				Value (N=36), n (%)
**Type**				
	Consensus statement		14 (39)	
	Guideline		19 (53)	
	Standard		3 (8)	
**Research objective**				
	**Disease**			
		Diabetic retinopathy	2 (6)	
		Mammary gland	1 (3)	
		Large intestinal tumor	1 (3)	
		Rectal cancer	1 (3)	
		Esophageal cancer	1 (3)	
		Glaucoma	1 (3)	
		Pulmonary nodules	2 (6)	
	AI^a^ reporting		3 (8)	
	Medical imaging or medical radiology		5 (14)	
	AI data acquisition, labeling, and calculation		14 (39)	
	AI ethics and governance		5 (14)	
**Application**				
	**Disease screening, diagnosis, and treatment**			
		Retinal screening	2 (6)	
		Breast disease screening	1 (3)	
		Esophageal cancer diagnosis and treatment	1 (3)	
		Colorectal tumor	2 (6)	
		Glaucoma auxiliary screening	1 (3)	
		Pulmonary nodules	2 (6)	
	AI intervention reporting guideline		3 (8)	
	AI image/radiology development and cooperation		5 (14)	
	**AI data or image application**			
		Digestive endoscopy	1 (3)	
		Central nervous system tumor	1 (3)	
		Local lesion of the liver	1 (3)	
		Colorectal cancer	1 (3)	
		Chest radiography	1 (3)	
		Eye disease image	1 (3)	
		Body composition assessment	1 (3)	
		Personalized medicine	1 (3)	
		Biomedical data set construction	1 (3)	
		Population-based health indicators	1 (3)	
		AI electrocardiogram	1 (3)	
		Pneumoconiosis	1 (3)	
		Pulmonary nodules on chest computed tomography	2 (6)	
	AI ethics governance and applications		5 (14)	
**Study area**				
	North America		5 (14)	
	Europe		11 (31)	
	Asia		19 (53)	
	Australia		1 (3)	
**Register**				
	Yes		4 (11)	
	No		32 (89)	

^a^AI: artificial intelligence.

### Trend of AI Guideline Application and Development

#### Classification 1: Disease Screening, Diagnosis, and Treatment

A total of 9 articles on guidelines and consensus statements described the application of AI in disease screening, diagnosis, and treatment. Two guideline articles were about AI screening of retinopathy, with one emphasizing the remote determination of retinopathy severity by AI using optical coherence tomography images [19] and the other emphasizing the urgent need to establish a unified standard for AI-assisted retinopathy screening and formulate relevant specifications and recommendations for an AI diagnosis platform in terms of hardware parameters, color fundus photography, equipment configuration, data acquisition and standards, database establishment, AI algorithm requirements, AI screening report content format, and clinical AI screening follow-up programs [20]. One guideline article was about AI screening of breast diseases, and the results indicated that AI CAD programs could be applied to 2D mammography and mastectomy, synthetic mammography, and personalized screening [21]. One guideline article was about color endoscopy technology for AI detection of colorectal tumor lesion characterization. The results indicated that high-definition endoscopes, color endoscopes or dye-based endoscopes, virtual color endoscopes, and additional devices can be used for the detection of colorectal tumors [22]. In a guideline article, AI was used for the identification of rectal cancer, the preoperative assessment variables for rectal cancer staging were “T, N, CRM, and extramural vascular invasion (EMVI),” and AI was employed to complete the assessment of tumor staging [23]. One consensus statement article was about the application of AI in esophageal cancer diagnosis and treatment, which showed the data management, image feature extraction and feature screening requirements, model construction and validation, current status, and clinical application recommendations of AI in esophageal cancer diagnosis and treatment [24]. One guideline article was about the application of AI in glaucoma auxiliary screening, which formulated unified standards for data acquisition, algorithm model construction, hardware requirements, data set establishment and annotation, and an AI screening scheme for an AI glaucoma auxiliary screening system [25]. One guideline article was about the diagnosis and treatment of lung nodules. It recommended that 3D visualization of lung nodules by AI could be used for localization and surgical planning, noting that 3D visualization analysis for patients who had been initially diagnosed with lung nodules and required surgery could provide greater support for accurate preoperative diagnosis, precise intraoperative surgery, rapid patient recovery, and maximum benefit [26]. One consensus statement article looked at the current state and limitations of pulmonary nodule diagnosis, summarized the role of AI in pulmonary nodule identification and the differential diagnosis of benign and malignant pulmonary nodules, and highlighted the role of AI in the pathological classification prediction of pulmonary nodules, which was useful for surgical planning and surgical safety and accuracy [27].

#### Classification 2: AI Intervention Trial Reporting Guidelines

Two guideline articles sought to standardize the reporting of clinical trials and protocols that involve AI interventions, such as CONSORT-AI [28] and the SPIRIT-AI extension [29]. They standardized the intervention integration environment, data processing considerations, and error case analysis procedure of AI interventions in clinical trials. A standard article set forth the minimum information required for medical AI reports, which can be used to help the design and implementation of medical AI models, promote clinical decision-making, and manage issues related to accuracy and bias [30].

#### Classification 3: AI Imaging Development and Collaboration

There were 3 guideline articles [31-33] and 2 consensus statement articles [34,35] for AI development in medical images and collaboration. One guideline article offered up-to-date application suggestions for clinical practitioners, researchers, scholars, and users of clinical imaging and therapeutic radiotherapy services. It illustrated that radiotherapy practice, education, and research must be gradually adapted to AI development to maximize the benefits of AI technology [31]. A guideline article provided suggestions on model performance evaluation for medical image segmentation and suggestions on the evaluation index and measurement [32]. Many commercial solutions based on AI technology are on the market, and another guideline article suggested that radiologists should focus on practical problems to be considered when evaluating AI solutions for medical imaging, allowing all stakeholders to discuss with manufacturers and make an informed decision on whether to purchase AI commercial solutions for imaging applications [33].

One consensus statement article presented the development of AI molecular imaging in China and proposed expert consensus on promoting AI application and organizing implementation [35]. One consensus statement article was about the practices for AI image development and evaluation in dermatology, and included a project list that had been developed for AI skin image reports, establishing a comprehensive standard for report development and performance evaluation [34].

#### Classification 4: AI Data Application

Ten consensus statement articles, 3 guideline articles, and 1 standard article reported on the application rules for AI data acquisition, annotation, calculation, and quality control. One consensus statement article presented usage standards on AI data acquisition, annotation, storage, privacy protection, and data security for gastrointestinal endoscopy [36]. One consensus statement article presented the unified magnetic resonance imaging (MRI) data acquisition and AI image annotation rules for central nervous system tumors [37]. One consensus statement article established expert consensus on CT, MRI, and MRI hepatobiliary-specific contrast image annotation for focal liver lesions [38]. An expert consensus was also created for colorectal cancer CT or MRI based on several elements, such as colorectal cancer definition and image performance, annotation categories, methodologies, precautions, annotation principles, annotation requirements, annotators, and processes [39]. For chest radiography, an AI image quality control algorithm was created to establish the terminologies and definitions of image quality control, common image quality control problems, image quality factors, chest film posterior-anterior photography specifications, image evaluation criteria, and design principles [40]. For building an AI ophthalmology image database, specifications and recommendations were established for the quality evaluation of incoming data types, data information, data quality, informed consent, and data sharing [41].

According to a consensus statement article, upper abdominal MRI was appropriate for body composition analysis because of standardized data acquisition and evaluation, subject preparation, magnetic resonance (MR) parameter setting, and comprehensive evaluation of MR image quality for AI systems that aimed for automatic body composition quantification [42].

Experts from all areas conducted special research and in-depth discussion on how to label and control the chest digital radiography data of pneumoconiosis, and reached a consensus on the collection, screening, quality control, labeling content, labeling methods, labeling rules, labeling process, and quality judgment of chest digital radiography data of pneumoconiosis [43]. There was a consensus statement article that explained the labeling rules, labeling process, and quality control of lung nodule CT [44]. Another consensus statement article put forward the construction process of a chest CT lung nodule data set [45].

One guideline article discussed the most relevant computational models for personalized medicine in detail, defining specific challenges and providing applicable guidelines and recommendations for study design, data acquisition, and operation, as well as for model validation and clinical translation, and other research areas [46].

Based on 5 projects in different medical fields, a guideline article iteratively optimized the procedures for constructing data sets, and developed a set of guidelines and good practices that should be followed when constructing new medical data sets [47]. To improve population health information, another guideline article developed a structured interdisciplinary method for the study of population health indicators using health data and machine learning technology [48].

In order to effectively promote the research and development process of AI ECG diagnosis, a standard article formulated the “Application Standard of Artificial Intelligence Diagnosis of ECG in Sichuan Province (for Trial Implementation),” expounding the normal standard and early warning standard of ECG labeling [49].

#### Classification 5: AI Ethics Governance and Applications

Four guideline articles and one standard article reported the application rules for ethical governance of AI [50-54]. There was a guideline article that discussed researchers’ use of a system framework to identify the ethical consequences of various research designs and algorithm choices, and it took epilepsy as an example, discussed the expected clinical scenarios that illustrate unexpected ethical consequences, evaluated the failure points in each scenario, and provided practical suggestions for understanding and solving ethical problems in the early stage of method development [50]. One guideline article mentioned that the current consensus on “ethical AI” should carefully examine the moral norms that claim to respect human rights and the need to emphasize the possibility of expanding socioeconomic inequality. The governance of AI design, development, and deployment needs a strong human rights framework to protect the public from the threat of harmful applications [51]. One guideline article drafted the ethical principles of using AI and the standards of AI practice in clinical radiology [52]. The World Health Organization issued a guiding document on the ethics and governance of AI in the health field; analyzed various opportunities and challenges brought by AI; and put forward suggestions on the policies, principles, and practices of using AI in the health field in an ethical way, as well as the means to avoid abusing AI to harm human rights and legal obligations [53]. A standard article discussed the application standard system framework and typical application scenarios of medical and health AI from the perspectives of basic common standards, AI basic technology, technical risks, product and service standards, application practice standards in the medical and health fields, safety ethics, and application evaluation standards [54].

### Evaluation of Methodological Quality Using the AGREE II Instrument

In all 6 domains of the AGREE II instrument, the ICCs between the 4 reviewers were high (>0.7), indicating good general agreement between them.

On a 7-point Likert scale, the average overall assessment ratings for the 36 guidelines, consensus statements, and standards evaluated ranged from 2.2 to 5.5 (with 7 indicating that the item’s criteria had been fully met). Nineteen articles had an overall assessment score of 4.0 or above, while 5 articles had an overall assessment score of 5.0 or above.

Based on the AGREE II assessments, only 5 articles were approved without changes by the appraisers [22,27-29,53], and it was concluded that 31 articles required further methodological refinement.

Score details of AGREE II are presented in Multimedia Appendix 4.

#### Percentage Quality Assessment of a Scaled Domain

Scope and purpose scores ranged from 52.8% to 106.9% across all studies, with an overall average of 81.2%. Stakeholder involvement scores varied from 20.8% to 101.4%, with a 51.6% average. The average score for rigor of development was 39.8%, with scores ranging from 11.5% to 82.8%. The average score for presentation clarity was 73.0%, with scores ranging from 6.9% to 116.7%. Applicability scores ranged from 29.2% to 102.1%, with a 57.6% average. The average score for editorial independence was 70.9%, with scores ranging from 8.3% to 108.3%.

#### Purpose and Scope

The overall aims were defined clearly for each of the studies, and the health goals and predicted outcomes of the recommendations were presented. All of the health questions covered by each study were also adequately defined and the targeted groups were sufficiently described in all but 2 studies [21,24].

#### Stakeholder Involvement

All but 8 articles [33,43-45,47,49,51,54] provided extensive information about the individuals engaged in the production of the guidelines or consensus statements, including their names, field of expertise, institution, and geographic location. All but 7 articles [22,27-29,31,34,53] did not include patient values and preferences in their creation. In most cases, the intended users were explicitly defined, with medical specialists and different types of health care practitioners included. Regarding target users, some of the articles were ambiguous [19,47,49,51,54].

#### Rigor of Development

Seventeen articles [24,26,30,34-40,42-45,49,54], particularly consensus statement and standard articles, did not describe their research process in sufficient detail.

Six guideline articles [21,22,28,29,34,48] that provided extensive systematic search methodologies also included detailed criteria for evidence selection. Except for 20 articles [23,30,32-35,37,38,43-54], the strengths and limitations of evidence were acknowledged in the other included studies. All articles, except for 5 [30,35,49,51,52], fully detailed the approach for creating recommendations. The guideline articles that incorporated the procedure in their methodologies used different ways of developing suggestions, with expert consensus, in-depth discussion, and feedback over numerous meetings used by 14 articles [20,22,23,25,28,29,31,33,41,42,50-53].

Although depth was lacking, 16 guideline articles considered health benefits, side effects, and hazards during the development process, and detailed the potential benefits and hazards of following the advice. Fourteen articles [19-22,24,26-29,31,34,35,37-41,53] included an explicit relationship between the recommendations and the body of supporting data. Five guideline articles [22,28,29,52,53] featured explicit declarations of expert external evaluation before publication, but 31 articles did not. In addition, 29 articles did not include any criteria or mechanisms for future changes. Eight articles [19,21,22,28,29,31,52,53] indicated steps for updating.

#### Clarity of Presentation

All guideline articles, except for 1 [35], included recommendations that were explicit, straightforward, and immediately identifiable. Different options or hygiene issues were not listed in 3 guideline articles [21,35,38], and recommendations were not easy to identify in 4 guideline articles [21,35,37,38].

#### Applicability

Twenty-seven articles [19-29,31,33-42,44,46,48,50-53] mentioned the application’s facilitators and barriers in detail and offered guidance, resources, or both for putting the recommendations into action. Twenty-seven articles [19-29,31,33-42,46-48,50-53] took into account the potential resource consequences of implementing the recommendations. Twenty-two articles [19,22,23,27-29,32,33,35,38,39,42-53] included monitoring or auditing criteria for determining the effectiveness of their recommendations.

#### Independence

Twenty-five articles [19-21,23-26,28,29,31-34,36-41,44-46, 48,50,51] explicitly stated their editorial independence. Furthermore, while 27 articles [20-26,29-34,36-46,48,51,52] specified competing interests, they failed to completely explain what interests were taken into account and how they were gathered.

#### Subgroup Analysis of Guidelines, Consensus Statements, and Standards

The average assessment score of AGREE II for the 14 consensus statement articles was 4.0 (range 3.0-5.1; 7-point Likert scale). The average assessment score for the 19 guideline articles was 4.2 (range 2.9-5.5).

The evaluation percentage rate results were defined as high quality when the score was >70% (Table 2). For the 14 consensus statement articles, the average scores of AGREE II domains 1-6 were 77.1%, 50.1%, 38.5%, 63.0%, 53.4%, and 76.6%, respectively. This shows that domains 2 (stakeholder involvement), 3 (rigor of development), 4 (clarity of presentation), and 5 (applicability) all need to be improved.

For the 19 guideline articles, the average scores of AGREE II domains 1-6 were 83.6%, 55.4%, 44.1%, 78.9%, 64.1%, and 73.1%, respectively. This shows that domains 2 (stakeholder involvement), 3 (rigor of development), and 5 (applicability) need to be improved.

For the 3 standard articles, the average scores of AGREE II domains 1-6 were 84.7%, 34.7%, 18.2%, 81.9%, 36.1%, and 29.9%, respectively. This shows that domains 2 (stakeholder involvement), 3 (rigor of development), 5 (applicability), and 6 (independence) need to be improved.

**Table 2:** AGREE II (Appraisal of Guidelines for Research & Evaluation II) evaluation results of the articles involving consensus statements, guidelines, and standards.

Domain	Consensus statements (n=14)	Guidelines (n=19)	Standards (n=3)
Domain 1	77.1%	83.6%	84.7%
Domain 2	50.1%	55.4%	34.7%
Domain 3	38.5%	44.1%	18.2%
Domain 4	63.0%	78.9%	81.9%
Domain 5	53.4%	64.1%	36.1%
Domain 6	76.6%	73.1%	29.9%

#### Subgroup Analysis of Different Contents

The average scores of AGREE II were 4.5 (range 3.8-5.4) for articles involving disease screening, diagnosis, and treatment (classification 1); 4.6 (range 2.8-5.5) for articles involving AI intervention trial reporting guidelines (classification 2); 4.0 (range 3.0-4.7) for articles involving AI imaging development and collaboration (classification 3); 3.6 (range 2.4-4.5) for articles involving AI data application (classification 4); and 3.7 (range 2.2-5.3) for articles involving AI ethics governance (classification 5).

For articles in classification 1, the average scores of AGREE II domains 1-6 were 73.5%, 52.6%, 48.6%, 67.9%, 57.5%, and 73.6%, respectively, showing that domains 2 (stakeholder involvement), 3 (rigor of development), 4 (clarity of presentation), and 5 (applicability) need to be improved. For articles in classification 2, the average scores of AGREE II domains 1-6 were 81.5%, 74.1%, 55.2%, 73.1%, 60.4%, and 63.2%, respectively, showing that domains 3 (rigor of development), 5 (applicability), and 6 (independence) need to be improved. For articles in classification 3, the average scores of AGREE II domains 1-6 were 82.2%, 55.6%, 38.0%, 58.1%, 54.2%, and 92.5%, respectively, showing that domains 2 (stakeholder involvement), 3 (rigor of development), 4 (clarity of presentation), and 5 (applicability) need to be improved. For articles in classification 4, the average scores of AGREE II domains 1-6 were 83.1%, 44.4%, 31.1%, 74.2%, 53.3%, and 69.9%, respectively, showing that domains 2 (stakeholder involvement), 3 (rigor of development), 5 (applicability), and 6 (independence) need to be improved. For articles in classification 5, the average scores of AGREE II domains 1-6 were 88.3%, 52.5%, 40.9%, 93.6%, 71.9%, and 51.7%, respectively, showing that domains 2 (stakeholder involvement), 3 (rigor of development), and 6 (independence) need to be improved. The results are presented in Table 3.

**Table 3:** AGREE II (Appraisal of Guidelines for Research & Evaluation II) evaluation results of the articles involving consensus statements, guidelines, and standards according to content classification.

Domain	Classification^a^				
	1 (n=9)	2 (n=3)	3 (n=5)	4 (n=14)	5 (n=5)
Domain 1	73.5%	81.5%	82.2%	83.1%	88.3%
Domain 2	52.6%	74.1%	55.6%	44.4%	52.5%
Domain 3	48.6%	55.2%	38.0%	31.1%	40.9%
Domain 4	67.9%	73.1%	58.1%	74.2%	93.6%
Domain 5	57.5%	60.4%	54.2%	53.3%	71.9%
Domain 6	73.6%	63.2%	92.5%	69.9%	51.7%

^a^Classification 1 includes articles involving disease screening, diagnosis, and treatment; classification 2 includes articles involving AI intervention trial reporting guidelines; classification 3 includes articles involving AI imaging development and collaboration; classification 4 includes articles involving AI data application; and classification 5 includes articles involving AI ethics governance.

### Evaluation of Reporting Quality (RIGHT Statement)

Among the 35 individual items, 9 were reported more than 80% (1a, 1c, 4, 5, 6, 7a, 8a, 10a, and 13a) and 5 were reported between 60% and 80% (2, 3, 10b, 18b, and 19a). The mean domain reporting rates were as follows: section 1 (basic information), 79.2% (range 33.3%-100.0%); section 2 (background), 59.4% (range 25.0%-100.0%); section 3 (evidence), 42.8% (range 20.0%-80.0%); section 4 (recommendations), 37.1% (range 14.3%-85.7%); section 5 (review and quality assurance), 13.9% (range 0.0%-100.0%); section 6 (funding, declaration, and management of interest), 47.9% (range 0.0%-100.0%); and section 7 (other information), 35.2% (range 0.0%-100.0%).

Among the 35 RIGHT checklist items, compliance with item 6 (describe the aims of the guidelines and specific objectives, such as improvements in health indicators, quality of life, or cost savings) was the highest (100%), while compliance with items 7b (describe any subgroups that are given special consideration in the guideline), 12 (describe the approach used to assess the certainty of the body of evidence), and 13c (the strength of the recommendation and the quality of evidence supporting it) was the lowest (<10%).

Score details of RIGHT are presented in Multimedia Appendix 5.

#### Subgroup Analysis of Guidelines, Consensus Statements, and Standards

For the 14 consensus statement articles, the average rate of the RIGHT assessment was 45.5% (range 34.3%-62.9%). The rates for the sections ranged from 0.0% to 81.0%, and sections 2 (background), 3 (evidence), 4 (recommendations), 5 (review and quality assurance), 6 (funding, declaration, and management of interest), and 7 (other information) need to be improved.

For the 19 guideline articles, the average rate of the RIGHT assessment was 55.2% (range 37.1%-77.1%). The rates for the sections ranged from 26.3% to 82.5%, and sections 2 (background), 3 (evidence), 4 (recommendations), 5 (review and quality assurance), 6 (funding, declaration, and management of interest), and 7 (other information) need to be improved.

For the 3 standard articles, the average rate of the RIGHT assessment was 31.4% (range 25.7%-42.9%). The rates for the sections ranged from 0.0% to 54.2%, and all the sections need to be improved. Details are provided in Table 4.

**Table 4:** RIGHT (Reporting Items for Practice Guidelines in Healthcare) evaluation results of the articles involving consensus statements, guidelines, and standards.

Section	Consensus statements (n=19)	Guidelines (n=14)	Standards (n=3)
Section 1	81.0%	82.5%	50.0%
Section 2	54.5%	63.8%	54.2%
Section 3	38.6%	47.4%	33.3%
Section 4	28.4%	47.1%	14.3%
Section 5	0.0%	26.3%	0.0%
Section 6	50.0%	52.6%	8.3%
Section 7	23.8%	45.6%	22.2%

#### Subgroup Analysis by Content Classification

For articles involving disease screening, diagnosis, and treatment (classification 1), the average rate of the RIGHT assessment was 50.5% (range 40.0%-77.1%), and the average rates for sections 1-7 were 79.8%, 61.6%, 51.4%, 43.5%, 17.9%, 41.1%, and 35.7%, respectively, showing that sections 2 (background), 3 (evidence), 4 (recommendations), 5 (review and quality assurance), 6 (funding, declaration, and management of interest), and 7 (other information) need to be improved.

For articles involving AI intervention trial reporting guidelines (classification 2), the average rate was 55.2% (range 42.9%-62.9%), and the average rates for sections 1-7 were 88.9%, 54.2%, 40.0%, 27.0%, 66.7%, 75.0%, and 55.6%, respectively, showing that sections 2 (background), 3 (evidence), 4 (recommendations), 5 (review and quality assurance), and 7 (other information) need to be improved.

For articles involving AI imaging development and collaboration (classification 3), the average rate was 60.0% (range 45.7%-68.6%), and the average rates for sections 1-7 were 90.0%, 65.0%, 48.0%, 43.8%, 10.0%, 75.0%, and 66.7%, respectively, showing that sections 2 (background), 3 (evidence), 4 (recommendations), 5 (review and quality assurance), and 7 (other information) need to be improved.

For articles involving AI data application (classification 4), the average rate was 41.8% (range 25.7%-68.6%), and the average rates for sections 1-7 were 72.6%, 56.3%, 32.9%, 30.4%, 0.0%, 39.3%, and 19.0%, respectively, showing that sections 2 (background), 3 (evidence), 4 (recommendations), 5 (review and quality assurance), 6 (funding, declaration, and management of interest), and 7 (other information) need to be improved.

For articles involving AI ethics governance (classification 5), the average rate was 54.9% (range 25.7%-74.3%), and the average rates for sections 1-7 were 63.3%, 67.5%, 52.0%, 60.0%, 30.0%, 20.0%, and 60.0%, respectively, showing that all sections need to be improved. Details are provided in Table 5.

For 3 types of classifications of articles involving guidelines and consensus statements in this review (disease screening, diagnosis, and treatment; AI imaging development and collaboration; and AI data application), background, methodological design, sources and evaluation of evidence, formation method and strength of recommendations of evidence, and promotion and application of the guidelines of recommendations of evidence are key areas for improvement, with further clarity also needed on disclosure.

For guidelines and standards on the reporting of trials of AI interventions, the main areas for improvement are background, sources and evaluation of evidence, and formation method and strength of recommendations of evidence. For guidelines and standards on AI ethics governance and applications, the main areas for improvement are background, sources and evaluation of evidence, formation method and strength of recommendations of evidence, funding, declaration, management of interest, etc. The findings and recommendations for different types of articles are presented in Table 6.

**Table 5:** RIGHT (Reporting Items for Practice Guidelines in Healthcare) evaluation results of the articles involving consensus statements, guidelines, and standards according to content classification.

Section	Classification^a^				
	1 (n=9)	2 (n=3)	3 (n=5)	4 (n=14)	5 (n=5)
Section 1	79.8%	88.9%	90.0%	72.6%	63.3%
Section 2	61.6%	54.2%	65.0%	56.3%	67.5%
Section 3	51.4%	40.0%	48.0%	32.9%	52.0%
Section 4	43.5%	27.0%	43.8%	30.4%	60.0%
Section 5	17.9%	66.7%	10.0%	0.0%	30.0%
Section 6	41.1%	75.0%	75.0%	39.3%	20.0%
Section 7	35.7%	55.6%	66.7%	19.0%	60.0%

^a^Classification 1 includes articles involving disease screening, diagnosis, and treatment; classification 2 includes articles involving AI intervention trial reporting guidelines; classification 3 includes articles involving AI imaging development and collaboration; classification 4 includes articles involving AI data application; and classification 5 includes articles involving AI ethics governance.

**Table 6:** Summary of the findings and recommendations.

Variable	Guidelines	Consensus statements	Standards	Classification				
				1: Disease screening, diagnosis, and treatment	2: AI^a^ intervention trial reporting guideline	3: AI imaging development and collaboration	4: AI data quality evaluation	5: AI ethics governance and applications
Average AGREE II^b^ score	4.2	4.0	2.5	4.5	4.6	4.0	3.6	3.7
Areas of AGREE II in need of improvement (reporting rate <70%)	Domains 2 (stakeholder involvement), 3 (rigor of development), and 5 (applicability)	Domains 2 (stakeholder involvement), 3 (rigor of development), 4 (clarity of presentation), and 5 (applicability)	Domains 2 (stakeholder involvement), 3 (rigor of development), 4 (clarity of presentation), 5 (applicability), and 6 (independence)	Domains 2 (stakeholder involvement), 3 (rigor of development), 4 (clarity of presentation), and 5 (applicability)	Domains 3 (rigor of development), 5 (applicability), and 6 (independence)	Domains 2 (stakeholder involvement), 3 (rigor of development), 4 (clarity of presentation), and 5 (applicability)	Domains 2 (stakeholder involvement), 3 (rigor of development), 5 (applicability), and 6 (independence)	Domains 2 (stakeholder involvement), 3 (rigor of development), and 6 (independence)
Average RIGHT^c^ reporting quality rate	55.2%	45.5%	31.4%	50.5%	55.2%	60.0%	41.8%	54.9%
Areas of RIGHT in need of improvement (reporting rate <70%)	Sections 2 (background), 3 (evidence), 4 (recommendations), 5 (review and quality assurance), 6 (funding, declaration, and management of interest), and 7 (other information)	Sections 2 (background), 3 (evidence), 4 (recommendations), 5 (review and quality assurance), 6 (funding, declaration, and management of interest), and 7 (other information)	Sections 1 (basic information), 2 (background), 3 (evidence), 4 (recommendations), 5 (review and quality assurance), 6 (funding, declaration, and management of interest), and 7 (other information)	Sections 2 (background), 3 (evidence), 4 (recommendations), 5 (review and quality assurance), 6 (funding, declaration, and management of interest), and 7 (other information)	Sections 2 (background), 3 (evidence), 4 (recommendations), 5 (review and quality assurance), and 7 (other information)	Sections 2 (background), 3 (evidence), 4 (recommendations), 5 (review and quality assurance), and 7 (other information)	Sections 2 (background), 3 (evidence), 4 (recommendations), 5 (review and quality assurance), 6 (funding, declaration, and management of interest), and 7 (other information)	Sections 1 (basic information), 2 (background), 3 (evidence), 4 (recommendations), 5 (review and quality assurance), 6 (funding, declaration, and management of interest), and 7 (other information)
Comprehensive recommendations	To more clearly describe the topics and primary users (eg, technicians, clinicians, etc). To improve methodological design. To improve sources and evaluation of evidence. To improve the formation method and strength of recommendations of evidence. To improve the promotion and application of recommendations of evidence. To improve the disclosure and management of conflicts of interest of recommendations of evidence.	To more clearly describe the topics and primary users (eg, technicians, clinicians, etc). To improve methodological design. To improve sources and evaluation of evidence. To improve the formation method and strength of recommendations of evidence. To improve the promotion and application of recommendations of evidence. To improve the disclosure and management of conflicts of interest of recommendations of evidence.	To more clearly describe the topics and primary users (eg, technicians, clinicians, etc). To improve methodological design. To improve sources and evaluation of evidence. To improve the formation method and strength of recommendations of evidence. To improve the promotion and application of recommendations of evidence. To improve the disclosure and management of conflicts of interest of recommendations of evidence.	To more clearly describe the topics and primary users (eg, technicians, clinicians, etc). To improve methodological design. To improve sources and evaluation of evidence. To improve the formation method and strength of recommendations of evidence. To improve the promotion and application of recommendations of evidence. To improve the disclosure and management of conflicts of interest of recommendations of evidence.	To improve the disclosure and management of conflicts of interest of recommendations of evidence. To more clearly describe the topics and primary users (eg, technicians, clinicians, etc). To improve sources and evaluation of evidence. To improve the formation method and strength of recommendations of evidence.	To more clearly describe the topics and primary users (eg, technicians, clinicians, etc). To improve methodological design. To improve sources and evaluation of evidence. To improve the formation method and strength of recommendations of evidence. To improve the promotion and application of recommendations of evidence. To improve the disclosure and management of conflicts of interest of recommendations of evidence.	To more clearly describe the topics and primary users (eg, technicians, clinicians, etc). To improve methodological design. To improve sources and evaluation of evidence. To improve the formation method and strength of recommendations of evidence. To improve the promotion and application of recommendations of evidence. To improve the disclosure and management of conflicts of interest of recommendations of evidence.	To more clearly describe the topics and primary users (eg, technicians, clinicians, etc). To improve methodological design. To improve sources and evaluation of evidence. To improve the formation method and strength of recommendations of evidence. To improve the promotion and application of recommendations of evidence. To improve the disclosure and management of conflicts of interest of recommendations of evidence.

^a^AI: artificial intelligence.

^b^AGREE II: Appraisal of Guidelines for Research & Evaluation II.

^c^RIGHT: Reporting Items for Practice Guidelines in Healthcare.

## Discussion

### Summary of the Findings

In recent years, medical technology, AI technology, and their combined application have been rapidly developed. With the expansion of medical data, application of medical images, improvement of AI algorithm models, and optimization of software and hardware devices, more AI technologies have started to be applied in health care scenarios to assist in making decisions on diagnosis and treatment. More medical institutions, internet companies, and nascent AI companies have started to seek cooperation with each other and have vigorously developed medical AI products, and more hospitals have been actively involved in collaborative research projects on medical AI. As a result, the field of medical AI has attracted many top scholars in terms of guideline development and scientific research, and some guidelines, expert consensus statements, and standards have been published in international journals in the field of medical AI.

To ensure that health care practitioners make well-informed decisions about the use of AI and have access to more reliable evidence-based resources, this study presents a systematic review of 36 articles published in English and Chinese between 2019 and 2022, evaluating them for methodological and reporting quality.

This study included 14 consensus statement articles, 19 guideline articles, and 3 standard articles, which were classified into 5 categories based on their content: (1) Disease screening, diagnosis, and treatment; (2) AI intervention trial reporting guidelines; (3) AI imaging development and collaboration; (4) AI data application; and (5) AI ethics governance and applications.

The average scores from the assessment of methodological quality using the AGREE II tool ranged from 2.2 to 5.5 on a 7-point Likert scale. The mean reporting quality rate using the RIGHT tool was 49.4%, ranging from 25.7% to 77.1%. Guideline articles scored higher than consensus statement articles and standard articles. There were higher proportions for the classification of AI intervention trial reporting guidelines than for the other classifications. Domains 2, 3, and 5 of the methodological quality tool and sections 2, 3, 4, 5, 6, and 7 of the reporting quality tool are most in need of improvement.

### Recommendations for Improving the Quality of AI Guidelines, Consensus Statements, and Standards

The development of guidelines must adhere to a strict systematic technique. Strict criteria must be developed to assure the quality of the guidelines. The main phases for guidelines, consensus statements, and standards are essentially the same: subject selection, evidence synthesis, recommendation creation, peer review, publishing, implementation, and updating [55]. However, in the 36 included studies, the forming methods were not ideal. The methodological quality of these documents needs to be improved in several categories, particularly rigor of development, stakeholder involvement, applicability, and reporting quality (background, evidence, recommendations, review, quality assurance, funding, declaration, management of interest, and other information). However, basic information, including scope and purpose, is already of a good standard.

#### Background for AI Guidelines, Consensus Statements, and Standards

Based on the results of the RIGHT assessment, the topics in the articles involving guidelines and consensus statements need to be more clearly described; need to cover the medical problems that the AI would be applied to (eg, disease screening, diagnosis, etc), the aims and specific objectives (eg, how AI applications are regulated), and the principal objectives or any subgroups covered by the recommendations of the guidelines (eg, clinical practitioners, medical data, or a certain type of AI technology); and need to identify the primary users of the guidelines (eg, technicians, clinicians, etc).

#### Methodological Design for AI Guidelines, Consensus Statements, and Standards

Based on the stakeholder involvement and rigor of development domains in AGREE II and section 5 (review and quality assurance) in RIGHT, the guideline developer should determine the targeted objects, technology, and population, and consider their preferences or development status. A reasonable evidence selection process, such as a systematic review, survey, or voting, should also be determined by the guideline developer with clear criteria stated for picking evidence, conducting surveys, or voting. At the same time, the guideline’s external evaluation scheme, comprising the list of evaluation experts and the treatment process for evaluation opinions, should be determined. After the draft guideline is finalized, it should be sent to specialists in relevant fields for review and made publicly available on the internet for public comment. Finally, the collected opinions should be evaluated and used to amend the guideline, and a mechanism should be put in place for updating it.

#### Sources and Evaluation of Evidence for AI Guidelines, Consensus Statements, and Standards

Based on the rigor of development domain in AGREE II and section 3 (evidence) in RIGHT, there are several areas for improvement. This might include stating the key questions for the recommendations in PICOS (Patient/Population, Intervention, Comparison, Outcome, Study design) or other formats as appropriate and indicating whether the guideline is based on a new systematic review conducted specifically for the guideline. The entire process of reference retrieval, including period, database, keywords, etc, should be provided in detail for the systematic review. Evidence inclusion and exclusion criteria should be established and followed, and formal techniques or methodologies (such as the GRADE [Grading of Recommendations Assessment, Development and Evaluation] system) should be used to assess the strengths and limitations of the evidence.

#### Formation Method and Strength of Recommendations of Evidence for AI Guidelines, Consensus Statements, and Standards

Based on the rigor of development and clarity of presentation domains in AGREE II and sections 4 (recommendations) and 5 (review and quality assurance) in RIGHT, the guideline should include a full description of the process used to create the recommendations, including how consensus was established and obtained. The guideline should also clearly state the grade of evidence, recommendations, and intensity of any suggestions, as determined by methods such as GRADE. The benefits and hazards of using AI in the medical profession should be explored, and there should be an explicit link between the recommendations and the supporting research. If the users of AI guidelines are intended to include different populations, or cost and resource implications are considered, the different advice for management of the AI issue should be clearly presented. The document should also indicate how the draft guideline underwent review and how this was used to inform the quality assurance process described in the methodological design.

#### Promotion and Application of the Guidelines of Recommendations of Evidence for AI Guidelines, Consensus Statements, and Standards

Based on the applicability domain in AGREE II and section 7 (access, suggestions for further research, limitations) in RIGHT, the guideline’s promotion and implementation strategy, which includes the target people, objects, technology, and data, should be developed. The potential benefits and hazards of implementing the recommendations, as well as the expenses and resources required to promote the guideline should be included, along with information on how the recommendations can be implemented, and the parameters and methods used by AI applications. Moreover, mechanisms should be put in place to ensure responsibility and accountability for AI systems and their outcomes. Furthermore, an adequate accessible redress should be ensured, especially in critical applications [56]. Finally, because AI is developing so quickly, it is important to specify where the guideline and its related materials can be found, as well as the limitations and suggestions for further research, and plans for keeping the document up to date.

#### Disclosure and Management of Conflicts of Recommendations of Evidence for AI Guidelines, Consensus Statements, and Standards

Based on the editorial independence section in AGREE II and section 6 (funding, declaration, and management of interest) in RIGHT, guideline documents should pay attention to providing precise information on conflicts of interest. For example, each team member should submit a conflict of interest disclosure statement, which can be used as a reference for guideline developers and include a declaration of employment, research grants, and other research support, among other things.

### Trends in the Application of AI in Health Care

In addition to the 5 AI application classifications identified in this review, AI applications in health care currently include intelligent guidance to patients to find the most appropriate departments and experts for consultation, clinical intelligence to assist in decision-making, early warning of clinical behavior, patient prognosis analysis, intelligent rationalization of treatment recommendations, and prediction of personal health or disease status. In the future, AI may also be used for more profound therapeutic areas, such as brain-machine interfaces (also known as brain-machine fusion perception), and to reconstruct special senses (eg, vision) and motor functions in paralyzed patients.

### Possible Challenges of AI

The 14 studies identified for classification 4 (AI data application) showed that data, arithmetic power, and algorithms are 3 core elements of AI, bringing new challenges for the implementation of AI in health care. The challenges of data include data quality, data annotation, data storage, data security, etc. To improve the learning efficiency of AI applications, a large amount of data annotation work is necessary, giving rise to more relevant guidelines and expert consensus statements. Due to the special nature of health care and health care systems, application system standards within different countries, regions, and hospitals are not uniform, making data collection irregular and imperfect. The challenges of massive data governance, technical robustness, and safety will also become increasingly important factors affecting the implementation of AI products, along with ethical approval, human oversight, privacy, transparency, nondiscrimination and fairness, societal well-being, and costs as important influencing factors [56]. This means that there is a great need for higher quality and more instructive guidelines to address a range of challenges.

### Future Research Directions for AI

Although the development of AI still faces many challenges, countries and industries are increasing their investment in AI applications owing to the significant potential advantages of AI technology in improving productivity, reducing costs, and improving service quality. The rapid year-on-year growth in the number of scientific and technical papers published on medical AI in recent years indicates that AI has also become a key research area of interest for experts and scholars. Research directions include deep learning, machine learning, biomedical engineering, automation, oncology, complementary diagnosis, and adjuvant therapy. In the future, increasing research evidence on medical AI will emerge, which will be more helpful for the development of AI guidelines, writing authoritative guidelines for more types of AI health issues, and making more standardized guidance recommendations.

### Innovation

To our knowledge, this systematic review is the first to use the AGREE II and RIGHT tools to evaluate AI guidelines, consensus statements, and standards. We reviewed and summarized articles involving international guidelines, consensus statements, and standards on the use of AI in health care published in recent years, as well as the main research directions. We also provide suggestions for methodological and reporting quality improvement for different types of documents. The rapid development of AI technology will see it being increasingly widely used in various fields, such as medical imaging, disease screening, and data learning, and this paper has also discussed future development trends, benefits, and potential hazards of AI applications in health care. We hope that it provides a scientific research and application reference for colleagues involved with AI in health care, and will help improve the quality and reporting of medical AI guidelines and provide a much needed foundation for improvements in the quality of research and practice [6].

### Limitations

Only 7 Chinese and English databases were included in the search strategy, and finally, only English and Chinese articles involving guidelines, consensus statements, and standards were included, which may cause limitations owing to restricted research sources and languages.

An important limitation of this systematic review is that it relied on studies published in few journals with high impact factors. Thus, there are disparities between some of the guidelines and others in terms of quality and authority, and the findings may not be fully representative of AI guidelines, consensus statements, and standards published around the world. Moreover, the articles included in this paper were considered as articles involving guidelines, consensus statements, or standards according to the definitions by the authors and journals themselves. Thus, the authority of the definitions may be limited owing to differences in quality and differences among the authors and journals.

Furthermore, we found that some items in the AGREE II and RIGHT tools are not fully applicable for evaluating medical guidelines related to AI, particularly those that use expert consensus statements and standards. As the clinical content of guidelines, consensus statements, and standards was not evaluated, no conclusions concerning the clinical appropriateness of the recommendations could be reached.

### Conclusions

Our systematic review identified 36 articles involving guidelines, consensus statements, and standards on the application of AI in health care. The main areas for the development and application of AI guidelines are disease screening and diagnosis, reporting of trials of AI interventions, AI image development and cooperation, AI data application, and AI ethics governance and applications. The application of AI in health care was generally encouraged in these articles, including the development of more standardized and standard algorithms, quality control of AI data, and clinical application of AI data for certain diseases. However, the quality of the included articles that we identified was not uniform, and there were differences in the methodological and reporting quality of guidelines for different research content. Most of the deficiencies were concentrated in domains 2, 3, and 5 of the AGREE II tool for methodological quality and sections 2, 3, 4, 5, 6, and 7 of the RIGHT tool for reporting quality.

Health care providers face challenges in gaining knowledge about the safe and effective use of AI. If the suggestions made for methodological and reporting quality improvements are followed, we believe that health care providers will have better access to higher quality guidance. This will be important if AI meets its potential for more powerful data induction and learning capabilities, which could significantly improve the application capabilities of medical imaging, disease screening, and diagnosis. We recommend that AI guidelines be further standardized in the future to improve the ability of AI deep learning and the ability of medical structured data service and sharing, and to strengthen the collection and fusion analysis of multicenter and multimodal medical data, allowing practitioners and scholars to cooperate in the best way to promote scientific research and clinical application.

## Appendix

Multimedia Appendix 1 Search strategies of the databases.

Multimedia Appendix 2 PRISMA (Preferred Reporting Items for Systematic Reviews and Meta-Analyses) 2020 checklist.

Multimedia Appendix 3 Characteristics of the included guidelines and consensus statements.

Multimedia Appendix 4 Score details of AGREE II (Appraisal of Guidelines for Research & Evaluation II).

Multimedia Appendix 5 Score details of RIGHT (Reporting Items for Practice Guidelines in Healthcare).

## Abbreviations

AGREE II: Appraisal of Guidelines for Research & Evaluation II
AI: artificial intelligence
CT: computed tomography
ECG: electrocardiography
GRADE: Grading of Recommendations Assessment, Development and Evaluation
ICC: intraclass correlation coefficient
MR: magnetic resonance
MRI: magnetic resonance imaging
PRISMA: Preferred Reporting Items for Systematic Reviews and Meta-Analyses
RIGHT: Reporting Items for Practice Guidelines in Healthcare

## Data Availability

All data needed to evaluate the conclusions in the paper are present in the paper and the multimedia appendices.
