# Correction: Guidelines, Consensus Statements, and Standards for the Use of Artificial Intelligence in Medicine: Systematic Review

**DOI:** 10.2196/55596

**Published:** 2023-12-21

**Authors:** Ying Wang, Nian Li, Lingmin Chen, Miaomiao Wu, Sha Meng, Zelei Dai, Yonggang Zhang, Mike Clarke

**Affiliations:** 1 Department of Medical Administration, West China Hospital, Sichuan University Chengdu China; 2 Department of Anesthesiology, National Clinical Research Center for Geriatrics, West China Hospital, Sichuan University Chengdu China; 3 Department of General Practice, National Clinical Research Center for Geriatrics, International Medical Center, West China Hospital, Sichuan University Chengdu China; 4 Department of Operation Management, West China Hospital, Sichuan University Chengdu China; 5 Department of Radiation Oncology, Cancer Center and State Key Laboratory of Biotherapy, West China Hospital, Sichuan University Chengdu China; 6 Department of Periodical Press, National Clinical Research Center for Geriatrics, Chinese Evidence-based Medicine Center, Nursing Key Laboratory of Sichuan Province, West China Hospital, Sichuan University Chengdu China; 7 Northern Ireland Methodology Hub, Queen's University Belfast Belfast United Kingdom

In “Guidelines, Consensus Statements, and Standards for the Use of Artificial Intelligence in Medicine: Systematic Review” (J Med Internet Res 2023;25:e46089) the authors noted that one phrase needed to be revised.

Under “Acknowledgments”, the following phrase:

The study was supported by the National Natural Science Foundation of China (grant number 82004213) and the Project of Sichuan Provincial Department of Science and Technology (number 2021YFH0191)

has been replaced by:

The study was supported by the National Natural Science Foundation of China (82174227, 82004213) and the Project of Sichuan Provincial Department of Science and Technology (2021YFH0191, 2022YFQ0069, 2023YFQ0071).

The correction will appear in the online version of the paper on the JMIR Publications website on December 22, 2023 together with the publication of this correction notice. Because this was made after submission to PubMed, PubMed Central, and other full-text repositories, the corrected article has also been resubmitted to those repositories.

