# Supplementary material for: Guidelines, Consensus Statements, and Standards for the Use of Artificial Intelligence in Medicine: Systematic Review
Source: J Med Internet Res. 2023 Nov 22;25:e46089. doi: 10.2196/46089 (PMC10701655; doi:10.2196/46089)
Supplement: Multimedia Appendix 1 [file jmir_v25i1e46089_app1.docx]

# Multimedia Appendix 1. Search strategies of the databases.

### Table S1. Search strategy of CNKI (2130)

| STEPS | Search Terms (in Chinese) |
| --- | --- |
| 1 | (TKA = Artificial Intelligence OR Machine Learning) AND (TKA = Consensus OR Guidelines OR Standards) AND (TKA = Medical OR Medicine OR Health) |

### Table S2. Search strategy of VIP (60)

| STEPS | Search Terms (in Chinese) |
| --- | --- |
| 1 | (Title or keyword = Artificial Intelligence OR Machine Learning) AND (Title or keyword = Consensus OR Guidelines OR Standards) AND (Title or keyword = Medical OR Medicine OR Health) |

### Table S3. Search strategy of Wanfang (1335)

| STEPS | Search Terms (in Chinese) |
| --- | --- |
| 1 | (Topic = Artificial Intelligence OR Machine Learning) AND (Topic = Consensus OR Guidelines OR Standards) AND (Topic = Medical OR Medicine OR Health) |

### Table S4. Search strategy of Sinomed (501)

| STEPS | Search Terms (in Chinese) |
| --- | --- |
| 1 | ( "Artificial Intelligence"[Common Field] OR "Machine Learning"[Common Field]) AND( "Consensus"[Common Field] OR "Guidelines"[Common Field] OR "Standard"[Common Field]) AND( "Medical" [Common Field] OR "Medical" [Common Field] OR "Health" [Common Field]) |

### Table S5. Search strategy of PubMed (1976)

| STEPS | | Search Terms |
| --- | --- | --- |
| 1 | (artificial intelligence[Title/Abstract]) OR (machine learning[Title/Abstract]) | |
| 2 | (guideline[Title/Abstract]) OR (consensus[Title/Abstract]) OR (standard[Title/Abstract]) | |
| 3 | (medicine[Title/Abstract]) OR (medical[Title/Abstract]) OR (healthcare[Title/Abstract]) OR (health[Title/Abstract]) | |
| 4 | #1 AND #2 AND #3 | |

### Table S6. Search strategy of Web of Science (4546)

| STEPS | | Search Terms |
| --- | --- | --- |
| 1 | ((TS=(artificial intelligence OR machine learning)) AND TS=(guideline OR consensus OR standard)) AND TS=(medicine OR medical OR healthcare OR health) | |

### Table S7. Search strategy of Embase (2326)

| STEPS | Search Terms |
| --- | --- |
| 1 | ((artificial intelligence OR machine learning) AND (guideline OR consensus OR standard) AND (medicine OR medical OR healthcare OR health)).ab. |
