# Supplementary material for: Guidelines, Consensus Statements, and Standards for the Use of Artificial Intelligence in Medicine: Systematic Review
Source: J Med Internet Res. 2023 Nov 22;25:e46089. doi: 10.2196/46089 (PMC10701655; doi:10.2196/46089)
Supplement: Multimedia Appendix 2 [file jmir_v25i1e46089_app2.docx]

# Multimedia Appendix 2. PRISMA (Preferred Reporting Items for Systematic Reviews and Meta-Analyses) 2020 checklist.

| **Section and Topic** | **Item #** | **Checklist item** | **Location where item is reported** |
| --- | --- | --- | --- |
| **TITLE** | | |  |
| Title | 1 | Identify the report as a systematic review. | Page 1, title |
| **ABSTRACT** | | |  |
| Abstract | 2 | See the PRISMA 2020 for Abstracts checklist. | Page 1, abstract |
| **INTRODUCTION** | | |  |
| Rationale | 3 | Describe the rationale for the review in the context of existing knowledge. | Page 1,  The first paragraph of the Introduction |
| Objectives | 4 | Provide an explicit statement of the objective(s) or question(s) the review addresses. | Page1-2,  The second paragraph of the Introduction |
| **METHODS** | | |  |
| Eligibility criteria | 5 | Specify the inclusion and exclusion criteria for the review and how studies were grouped for the syntheses. | Page 2,  Inclusion and exclusion criteria  Page 3,  Systematic review and statistical analysis |
| Information sources | 6 | Specify all databases, registers, websites, organisations, reference lists and other sources searched or consulted to identify studies. Specify the date when each source was last searched or consulted. | Page 2,  Search strategies |
| Search strategy | 7 | Present the full search strategies for all databases, registers and websites, including any filters and limits used. | Appendix 1: Search strategy |
| Selection process | 8 | Specify the methods used to decide whether a study met the inclusion criteria of the review, including how many reviewers screened each record and each report retrieved, whether they worked independently, and if applicable, details of automation tools used in the process. | Page 2,  Study Selection and Data Extraction, |
| Data collection process | 9 | Specify the methods used to collect data from reports, including how many reviewers collected data from each report, whether they worked independently, any processes for obtaining or confirming data from study investigators, and if applicable, details of automation tools used in the process. | Page 2-3,  Data extraction, Evaluation of the quality |
| Data items | 10a | List and define all outcomes for which data were sought. Specify whether all results that were compatible with each outcome domain in each study were sought (e.g. for all measures, time points, analyses), and if not, the methods used to decide which results to collect. | Page 2-3,  Data extraction, Evaluation of the quality |
|  | 10b | List and define all other variables for which data were sought (e.g. participant and intervention characteristics, funding sources). Describe any assumptions made about any missing or unclear information. | Page 2-3,  Data extraction, Evaluation of the quality |
| Study risk of bias assessment | 11 | Specify the methods used to assess risk of bias in the included studies, including details of the tool(s) used, how many reviewers assessed each study and whether they worked independently, and if applicable, details of automation tools used in the process. | Page 2-3, Evaluation of the quality |
| Effect measures | 12 | Specify for each outcome the effect measure(s) (e.g. risk ratio, mean difference) used in the synthesis or presentation of results. | Page 2-3, Evaluation of the quality |
| Synthesis methods | 13a | Describe the processes used to decide which studies were eligible for each synthesis (e.g. tabulating the study intervention characteristics and comparing against the planned groups for each synthesis (item #5)). | Page 3,  Systematic review and statistical analysis |
|  | 13b | Describe any methods required to prepare the data for presentation or synthesis, such as handling of missing summary statistics, or data conversions. | Page 2,  Data extraction |
|  | 13c | Describe any methods used to tabulate or visually display results of individual studies and syntheses. | Page 3,  Systematic review and statistical analysis |
|  | 13d | Describe any methods used to synthesize results and provide a rationale for the choice(s). If meta-analysis was performed, describe the model(s), method(s) to identify the presence and extent of statistical heterogeneity, and software package(s) used. | Page 3,  Systematic review and statistical analysis |
|  | 13e | Describe any methods used to explore possible causes of heterogeneity among study results (e.g. subgroup analysis, meta-regression). | Page 3,  Systematic review and statistical analysis |
|  | 13f | Describe any sensitivity analyses conducted to assess robustness of the synthesized results. | Not involved |
| Reporting bias assessment | 14 | Describe any methods used to assess risk of bias due to missing results in a synthesis (arising from reporting biases). | Page 2,  Data extraction |
| Certainty assessment | 15 | Describe any methods used to assess certainty (or confidence) in the body of evidence for an outcome. | Page 2-3,  Evaluation of the quality |
| **RESULTS** | | |  |
| Study selection | 16a | Describe the results of the search and selection process, from the number of records identified in the search to the number of studies included in the review, ideally using a flow diagram. | Page 4,  Fig. 1 |
|  | 16b | Cite studies that might appear to meet the inclusion criteria, but which were excluded, and explain why they were excluded. | Page 4,  Fig. 1 |
| Study characteristics | 17 | Cite each included study and present its characteristics. | Page 5-10,  Characteristics of included guidelines |
| Risk of bias in studies | 18 | Present assessments of risk of bias for each included study. | Not involved |
| Results of individual studies | 19 | For all outcomes, present, for each study: (a) summary statistics for each group (where appropriate) and (b) an effect estimate and its precision (e.g. confidence/credible interval), ideally using structured tables or plots. | Page 11-12, Table 2.  Appendix 2: Score details of AGREE II(Table S8-S9).  Appendix 3: Score details of RIGHT(Table S10). |
| Results of syntheses | 20a | For each synthesis, briefly summarise the characteristics and risk of bias among contributing studies. | Page 12-15,  Trend of AI guidelines application and development |
|  | 20b | Present results of all statistical syntheses conducted. If meta-analysis was done, present for each the summary estimate and its precision (e.g. confidence/credible interval) and measures of statistical heterogeneity. If comparing groups, describe the direction of the effect. | Page 15-20,  Evaluation of the methodological quality using the AGREE II instrument, Evaluation of reporting quality (RIGHT Statement) |
|  | 20c | Present results of all investigations of possible causes of heterogeneity among study results. | Page 15-20,  Evaluation of the methodological quality using the AGREE II instrument, Evaluation of reporting quality (RIGHT Statement) |
|  | 20d | Present results of all sensitivity analyses conducted to assess the robustness of the synthesized results. | Page 15-20,  Evaluation of the methodological quality using the AGREE II instrument, Evaluation of reporting quality (RIGHT Statement) |
| Reporting biases | 21 | Present assessments of risk of bias due to missing results (arising from reporting biases) for each synthesis assessed. | Not involved |
| Certainty of evidence | 22 | Present assessments of certainty (or confidence) in the body of evidence for each outcome assessed. | Page 15,  Evaluation of the methodological quality using the AGREE II instrument |
| **DISCUSSION** | | |  |
| Discussion | 23a | Provide a general interpretation of the results in the context of other evidence. | Page 20-21, Summary of findings |
|  | 23b | Discuss any limitations of the evidence included in the review. | Page 21,  Limitations |
|  | 23c | Discuss any limitations of the review processes used. | Page 21,  Limitations |
|  | 23d | Discuss implications of the results for practice, policy, and future research. | Page 25-27,  Trends in the application of AI in health care, Future Research Direction for AI, Innovation |
| **OTHER INFORMATION** | | |  |
| Registration and protocol | 24a | Provide registration information for the review, including register name and registration number, or state that the review was not registered. | Page 1,  Trial Registration |
|  | 24b | Indicate where the review protocol can be accessed, or state that a protocol was not prepared. | Page 1,  Trial Registration |
|  | 24c | Describe and explain any amendments to information provided at registration or in the protocol. | Not involved |
| Support | 25 | Describe sources of financial or non-financial support for the review, and the role of the funders or sponsors in the review. | Page 28,  Funding, Author contributions |
| Competing interests | 26 | Declare any competing interests of review authors. | Page 28,  Conflicts of Interest |
| Availability of data, code and other materials | 27 | Report which of the following are publicly available and where they can be found: template data collection forms; data extracted from included studies; data used for all analyses; analytic code; any other materials used in the review. | Page 28,  Data and materials availability |
