# Supplementary material for: Guidelines, Consensus Statements, and Standards for the Use of Artificial Intelligence in Medicine: Systematic Review
Source: J Med Internet Res. 2023 Nov 22;25:e46089. doi: 10.2196/46089 (PMC10701655; doi:10.2196/46089)
Supplement: Multimedia Appendix 3 [file jmir_v25i1e46089_app3.docx]

**Multimedia Appendix 3.** Characteristics of the included guidelines and consensus statements.

| No. | Author, year | Type | Region | Country | Major publishing organization | Journal/book series | Number of pages | Number of references | Register | Development methods |
| --- | --- | --- | --- | --- | --- | --- | --- | --- | --- | --- |
| 1 | Boucher et al [19], 2020 | Guideline | North America | Canada | Canadian Retina Research Network (CR2N) TELE-Retina Steering Committee | Canadian Journal of Ophthalmology | 11 | 73 | No | Based on existing evidence plus actual screening |
| 2 | Yuan et al [20], 2019 | Guideline | Asia | China | Intelligent Ophthalmology Group of Intelligent Medicine Special Committee of the China Medical Education Association | Chinese Journal of Experimental Ophthalmology | 6 | 19 | Yes | Based on consensus |
| 3 | Thomassin-Naggara et al [21], 2019 | Consensus statement | Europe | France | French Society of Radiology (SFR) and Commission on Radiology (CERF) | Diagnostic and Interventional Imaging | 14 | 54 | No | Literature review plus conference |
| 4 | Bisschops et al [22], 2019 | Guideline | Europe | Belgium | European Society for Gastrointestinal Endoscopy (ESGE) | Endoscopy | 25 | 252 | No | Standard guideline development method |
| 5 | Gao et al [23], 2021 | Guideline | Asia | China | Affiliated Hospital of Qingdao University | Chinese Medical Journal (English) | 3 | 17 | Yes | Based on consensus |
| 6 | Shan et al [24], 2021 | Consensus statement | Asia | China | Interventional Medicine Center Branch of the Chinese Hospital Association | Chinese Electronic Journal of Interventional Radiology | 12 | 80 | No | Based on consensus |
| 7 | Wang et al [25], 2020 | Guideline | Asia | China | Glaucoma Group of Ophthalmology Branch of the Chinese Medical Association | Chinese Journal of Ophthalmology | 10 | 33 | No | Based on consensus |
| 8 | Hu et al [26], 2019 | Consensus statement | Asia | China | Thoracic Surgery Professional Committee of the China Medical Education Association | Chinese Journal of Thoracic and Cardiovascular Surgery | 7 | 26 | No | Based on consensus |
| 9 | Long et al [27], 2022 | Consensus statement | Asia | China | Thoracic Surgery Committee, Department of Simulated Medicine, Wu Jieping Medical Foundation | Chinese Journal of Lung Cancer | 7 | 60 | No | Based on consensus |
| 10 | Liu et al [28], 2020 | Guideline | Europe | UK^a^ | University of Birmingham | Lancet Digital Health | 12 | 63 | No | Literature review plus expert consultation |
| 11 | Rivera et al [29], 2020 | Guideline | Europe | UK | University of Birmingham | Lancet Digital Health | 12 | 52 | No | Literature review plus Delphi plus expert consultation |
| 12 | Hernandez-Boussard et al [30], 2020 | Standard | North America | US | Department of Medicine, Stanford University | Journal of the American Medical Informatics Association | 5 | 26 | No | Unspecified |
| 13 | Malamateniou et al [31], 2021 | Guideline | Europe | UK | University of London | Radiography (London) | 11 | 31 | No | Literature review plus expert consultation |
| 14 | Muller et al [32], 2022 | Guideline | Europe | Germany | IT-Infrastructure for Translational Medical Research, University of Augsburg | BMC Research Notes | 7 | 34 | No | Unspecified |
| 15 | Omoumi et al [33], 2021 | Guideline | Europe | Switzerland | Department of Radiology, Lausanne University Hospital and University of Lausanne | European Radiology | 11 | 42 | No | Based on consensus |
| 16 | Daneshjou et al [34], 2022 | Consensus statement | North America | US^b^ | Memorial Sloan Kettering Cancer Center | JAMA Dermatology | 7 | 56 | No | Literature review plus expert consultation |
| 17 | Li [35], 2019 | Consensus statement | Asia | China | Chinese Medical Association Nuclear Medicine Branch Molecular Imaging Artificial Intelligence Working Committee | Chinese Journal of Nuclear Medicine and Molecular Imaging | 4 | 14 | No | Based on consensus |
| 18 | Li [36], 2020 | Consensus statement | Asia | China | Chinese Medical Doctor Association Digestive Endoscopy Artificial Intelligence Professional Committee | Chinese Journal of Digestive Endoscopy | 7 | 30 | No | Based on consensus |
| 19 | Hu et al [37], 2021 | Consensus statement | Asia | China | Internet plus Imaging Group of Radiology Branch of the Chinese Medical Doctor Association | International Journal of Medical Radiology | 7 | 18 | No | Based on consensus |
| 20 | Jin et al [38], 2020 | Consensus statement | Asia | China | Chinese Medical Association Radiology Branch Medical Imaging Big Data and Artificial Intelligence Working Committee | Chinese Journal of Radiology | 8 | 16 | No | Based on consensus |
| 21 | Jin et al [39], 2021 | Consensus statement | Asia | China | Medical Imaging Big Data and Artificial Intelligence Working Committee of Radiology Branch of the Chinese Medical Association | Chinese Journal of Radiology | 6 | 18 | No | Based on consensus |
| 22 | Fu et al [40], 2021 | Consensus statement | Asia | China | Imaging Technology Branch of the Chinese Medical Association and Jiufeng Medical | Chinese Medical Equipment | 3 | 0 | No | Based on consensus |
| 23 | Lin et al [41], 2019 | Guideline | Asia | China | “Belt and Road” International Alliance for Ophthalmic Artificial Intelligence R&D | Chinese Journal of Experimental Ophthalmology | 5 | 17 | Yes | Based on consensus |
| 24 | Lv et al [42], 2022 | Guideline | Asia | China | Beijing Friendship Hospital, Capital Medical University | Chinese Medical Journal | 3 | 6 | Yes | Based on consensus |
| 25 | Li et al [43], 2020 | Consensus statement | Asia | China | Standard Group of Chest Imaging and Occupational Diseases, Society of Medical Artificial Intelligence, Chinese Society of Biomedical Engineering | Environmental and Occupational Medicine | 7 | 5 | No | Based on consensus |
| 26 | Ren [44], 2019 | Consensus statement | Asia | China | Chinese Institute for Food and Drug Control, Cardiothoracic Group, Society of Radiology, Chinese Medical Association | Chinese Journal of Radiology | 7 | 14 | No | Based on consensus |
| 27 | Liu [45], 2021 | Consensus statement | Asia | China | Chinese Society of Radiology, Chinese Institute for Food and Drug Control | Chinese Journal of Radiology | 7 | 20 | No | Based on consensus |
| 28 | Collin et al [46], 2022 | Guideline | Europe | Denmark | Novo Nordisk Foundation Center for Protein Research, Faculty of Health and Medical Sciences, University of Copenhagen | Journal of Personalized Medicine | 24 | 144 | No | Unspecified |
| 29 | Cychnerski et al [47], 2021 | Guideline | Europe | Poland | Computer Vision and Artificial Intelligence Laboratory, Department of Computer Architecture, Faculty of Electronics, Telecommunications and Informatics, Gdańsk University of Technology | New Trends in Database and Information Systems, Communications in Computer and Information Science book series | 13 | 15 | No | Unspecified |
| 30 | Haneef et al [48], 2022 | Guideline | Europe | France | Department of Non-Communicable Diseases and Injuries | Archives of Public Health | 12 | 53 | No | Scientific literature review, identification of inspiring examples from European countries and developing the checklist of guideline content |
| 31 | Chengdu High-Tech Medical Association Cardiac Function Committee [49], 2021 | Standard | Asia | China | Cardiac Function Committee of Chengdu High-Tech Medical Association | Advances in Cardiology | 2 | 6 | No | Based on consensus |
| 32 | Chiang et al [50], 2021 | Guideline | North America | US | Department of Neurology and Weill Institute for Neurosciences, University of California | Neurology | 9 | 40 | No | Prospective case analysis was combined with a systematic literature review and improved Delphi methods |
| 33 | Fukuda-Parr et al [51], 2021 | Guideline | North America | US | The New School | Global Policy | 13 | 53 | No | Based on consensus |
| 34 | Kenny et al [52], 2021 | Guideline | Australia | Australia | Royal Brisbane and Women’s Hospital | Journal of Medical Imaging and Radiation Oncology | 9 | 23 | No | Based on consensus |
| 35 | WHO [53], 2021 | Guideline | Europe | Switzerland | Health Ethics and Governance Unit, Research for Health Department, Digital Health and Innovation Department, Division of the Chief Scientist | Chinese Medical Journal | 165 | 336 | No | Based on expert consensus and external review |
| 36 | Liang et al [54], 2021 | Standard | Asia | China | National Health Commission Statistical Information Center | Chinese Journal of Health Information Management | 5 | 16 | No | Unspecified |

^a^UK: United Kingdom.

^b^US: United States.
