# Supplementary material for: Guidelines, Consensus Statements, and Standards for the Use of Artificial Intelligence in Medicine: Systematic Review
Source: J Med Internet Res. 2023 Nov 22;25:e46089. doi: 10.2196/46089 (PMC10701655; doi:10.2196/46089)
Supplement: Multimedia Appendix 4 [file jmir_v25i1e46089_app4.docx]

# Multimedia Appendix 4. Score details of AGREE II (Appraisal of Guidelines for Research & Evaluation II).

### Table S1. Score details of AGREE II (articles 1-10)

| Reviewer | A | B | C | D | | A | B | C | D | | A | B | C | D | | A | B | C | D | | A | B | C | D | | A | B | C | D | | A | B | C | D | | A | B | C | D | | A | B | C | D | | A | B | C | D | |
| --- | --- | --- | --- | --- | --- | --- | --- | --- | --- | --- | --- | --- | --- | --- | --- | --- | --- | --- | --- | --- | --- | --- | --- | --- | --- | --- | --- | --- | --- | --- | --- | --- | --- | --- | --- | --- | --- | --- | --- | --- | --- | --- | --- | --- | --- | --- | --- | --- | --- | --- |
| **Section/Item** | Article 1 | | | | Article 2 | | | | | Article 3 | | | | | Article 4 | | | | | Article 5 | | | | | Article 6 | | | | | Article 7 | | | | | Article 8 | | | | | Article 9 | | | | | Article 10 | | | | |  |
| **Purpose and scope.** | | | | | | | | | | | | | | | | | | | | | | | | | | | | | | | | | | | | | | | | | | | | | | | | | |  |
| **1** | 5 | 5 | 5 | 6 | | 6 | 6 | 6 | 6 | | 5 | 5 | 5 | 6 | | 6 | 6 | 6 | 7 | | 6 | 6 | 6 | 6 | | 5 | 6 | 5 | 7 | | 6 | 6 | 7 | 7 | | 6 | 6 | 6 | 7 | | 6 | 7 | 6 | 7 | | 6 | 6 | 6 | 7 | |
| **2** | 4 | 5 | 5 | 5 | | 6 | 6 | 6 | 6 | | 5 | 6 | 5 | 5 | | 6 | 4 | 6 | 6 | | 6 | 4 | 6 | 5 | | 4 | 5 | 4 | 3 | | 6 | 6 | 6 | 6 | | 6 | 6 | 6 | 6 | | 6 | 7 | 6 | 6 | | 6 | 5 | 6 | 6 | |
| **3** | 6 | 4 | 4 | 6 | | 4 | 6 | 6 | 6 | | 2 | 4 | 2 | 4 | | 5 | 4 | 5 | 5 | | 4 | 5 | 4 | 6 | | 3 | 3 | 3 | 2 | | 4 | 5 | 5 | 5 | | 5 | 6 | 5 | 5 | | 6 | 7 | 6 | 7 | | 5 | 4 | 5 | 5 | |
| **Stakeholder involvement.** | | | | | | | | | | | | | | | | | | | | | | | | | | | | | | | | | | | | | | | | | | | | | | | | | |  |
| **4** | 5 | 4 | 4 | 5 | | 4 | 5 | 5 | 5 | | 6 | 6 | 6 | 6 | | 6 | 7 | 6 | 7 | | 4 | 5 | 4 | 5 | | 5 | 6 | 6 | 6 | | 5 | 6 | 5 | 5 | | 5 | 4 | 5 | 5 | | 6 | 7 | 7 | 6 | | 6 | 6 | 6 | 6 | |
| **5** | 1 | 1 | 1 | 1 | | 1 | 2 | 2 | 1 | | 1 | 1 | 1 | 1 | | 6 | 6 | 6 | 6 | | 2 | 1 | 2 | 1 | | 1 | 2 | 2 | 1 | | 1 | 2 | 1 | 1 | | 1 | 2 | 1 | 1 | | 6 | 7 | 7 | 6 | | 6 | 6 | 6 | 6 | |
| **6** | 1 | 4 | 3 | 3 | | 4 | 5 | 4 | 6 | | 1 | 4 | 1 | 6 | | 6 | 5 | 6 | 4 | | 5 | 5 | 5 | 6 | | 3 | 2 | 2 | 5 | | 4 | 5 | 5 | 5 | | 5 | 6 | 5 | 6 | | 6 | 7 | 7 | 6 | | 6 | 4 | 6 | 6 | |
| **Rigor of development.** | | | | | | | | | | | | | | | | | | | | | | | | | | | | | | | | | | | | | | | | | | | | | | | | | |  |
| **7** | 2 | 2 | 1 | 1 | | 1 | 2 | 2 | 1 | | 6 | 5 | 6 | 7 | | 6 | 6 | 6 | 6 | | 2 | 3 | 2 | 2 | | 1 | 1 | 1 | 1 | | 1 | 2 | 1 | 1 | | 1 | 2 | 1 | 1 | | 4 | 5 | 4 | 4 | | 7 | 6 | 7 | 7 | |
| **8** | 1 | 4 | 1 | 1 | | 4 | 5 | 5 | 5 | | 5 | 4 | 5 | 5 | | 6 | 5 | 6 | 6 | | 2 | 2 | 2 | 2 | | 2 | 2 | 2 | 1 | | 4 | 3 | 4 | 4 | | 4 | 5 | 4 | 4 | | 6 | 7 | 6 | 6 | | 6 | 5 | 7 | 7 | |
| **9** | 1 | 5 | 1 | 1 | | 4 | 5 | 5 | 4 | | 4 | 4 | 4 | 5 | | 7 | 4 | 6 | 6 | | 2 | 2 | 2 | 2 | | 3 | 2 | 2 | 2 | | 4 | 3 | 4 | 4 | | 4 | 5 | 4 | 1 | | 6 | 7 | 6 | 6 | | 6 | 4 | 6 | 6 | |
| **10** | 4 | 6 | 5 | 5 | | 6 | 6 | 6 | 5 | | 5 | 4 | 5 | 5 | | 7 | 6 | 6 | 6 | | 3 | 4 | 3 | 3 | | 2 | 2 | 2 | 2 | | 7 | 7 | 7 | 7 | | 6 | 6 | 6 | 1 | | 5 | 5 | 5 | 5 | | 6 | 6 | 6 | 6 | |
| **11** | 3 | 5 | 3 | 5 | | 4 | 5 | 5 | 4 | | 3 | 4 | 3 | 3 | | 6 | 6 | 6 | 6 | | 3 | 2 | 3 | 2 | | 4 | 5 | 5 | 5 | | 5 | 6 | 5 | 5 | | 5 | 6 | 5 | 5 | | 6 | 7 | 7 | 6 | | 4 | 4 | 4 | 3 | |
| **12** | 6 | 4 | 6 | 6 | | 4 | 5 | 5 | 4 | | 4 | 5 | 5 | 5 | | 6 | 5 | 6 | 6 | | 3 | 3 | 3 | 3 | | 3 | 4 | 4 | 2 | | 3 | 4 | 3 | 3 | | 4 | 5 | 4 | 5 | | 4 | 5 | 5 | 4 | | 6 | 4 | 4 | 6 | |
| **13** | 1 | 1 | 1 | 1 | | 2 | 3 | 3 | 2 | | 2 | 1 | 1 | 1 | | 4 | 7 | 7 | 7 | | 2 | 1 | 2 | 1 | | 1 | 1 | 1 | 1 | | 2 | 1 | 2 | 1 | | 2 | 4 | 2 | 2 | | 4 | 5 | 5 | 4 | | 5 | 4 | 4 | 6 | |
| **14** | 5 | 1 | 5 | 4 | | 2 | 3 | 3 | 2 | | 6 | 1 | 4 | 4 | | 6 | 6 | 6 | 6 | | 2 | 2 | 2 | 2 | | 1 | 1 | 1 | 1 | | 2 | 1 | 2 | 1 | | 2 | 3 | 2 | 1 | | 1 | 1 | 1 | 1 | | 4 | 5 | 5 | 6 | |
| **Clarity of presentation** | | | | | | | | | | | | | | | | | | | | | | | | | | | | | | | | | | | | | | | | | | | | | | | | | |  |
| **15** | 5 | 6 | 5 | 6 | | 6 | 6 | 6 | 6 | | 2 | 4 | 2 | 2 | | 5 | 5 | 5 | 5 | | 5 | 5 | 5 | 5 | | 2 | 2 | 2 | 1 | | 6 | 6 | 6 | 6 | | 5 | 5 | 5 | 5 | | 7 | 7 | 7 | 6 | | 6 | 6 | 6 | 7 | |
| **16** | 5 | 4 | 5 | 5 | | 4 | 5 | 4 | 5 | | 2 | 3 | 2 | 2 | | 6 | 3 | 6 | 5 | | 5 | 4 | 5 | 5 | | 2 | 1 | 2 | 1 | | 6 | 6 | 6 | 5 | | 5 | 5 | 5 | 4 | | 6 | 6 | 6 | 6 | | 5 | 4 | 5 | 4 | |
| **17** | 4 | 6 | 4 | 6 | | 6 | 6 | 6 | 6 | | 1 | 4 | 1 | 1 | | 5 | 5 | 5 | 6 | | 5 | 5 | 3 | 3 | | 1 | 1 | 1 | 1 | | 6 | 6 | 6 | 6 | | 5 | 5 | 5 | 5 | | 6 | 7 | 6 | 6 | | 6 | 6 | 6 | 7 | |
| **Applicability.** | | | | | | | | | | | | | | | | | | | | | | | | | | | | | | | | | | | | | | | | | | | | | | | | | |  |
| **18** | 5 | 6 | 5 | 5 | | 4 | 5 | 4 | 5 | | 4 | 5 | 5 | 5 | | 5 | 5 | 5 | 5 | | 4 | 4 | 4 | 4 | | 4 | 4 | 4 | 5 | | 5 | 6 | 5 | 3 | | 4 | 5 | 5 | 5 | | 6 | 5 | 5 | 6 | | 5 | 6 | 5 | 6 | |
| **19** | 1 | 4 | 1 | 1 | | 4 | 5 | 4 | 3 | | 1 | 6 | 6 | 1 | | 4 | 4 | 4 | 3 | | 4 | 5 | 3 | 3 | | 3 | 3 | 3 | 2 | | 4 | 6 | 4 | 3 | | 5 | 5 | 5 | 4 | | 3 | 2 | 3 | 3 | | 5 | 5 | 5 | 5 | |
| **20** | 6 | 4 | 6 | 6 | | 3 | 5 | 3 | 5 | | 4 | 6 | 6 | 6 | | 6 | 5 | 6 | 6 | | 4 | 4 | 4 | 4 | | 5 | 6 | 5 | 6 | | 4 | 5 | 4 | 4 | | 5 | 5 | 5 | 5 | | 5 | 4 | 5 | 4 | | 5 | 5 | 5 | 6 | |
| **21** | 5 | 4 | 5 | 6 | | **2** | **3** | **2** | **3** | | 2 | 3 | 3 | 2 | | 5 | 3 | 4 | 4 | | 6 | 2 | 6 | 6 | | 1 | 2 | 1 | 1 | | **2** | **3** | **2** | **2** | | **3** | **4** | **4** | **3** | | 5 | 5 | 5 | 5 | | 5 | 4 | 5 | 5 | |
| **Independence.** | | | | | | | | | | | | | | | | | | | | | | | | | | | | | | | | | | | | | | | | | | | | | | | | | |  |
| **22** | 6 | 4 | 6 | 7 | | 7 | 6 | 7 | 6 | | 6 | 7 | 7 | 7 | | 2 | 4 | 3 | 3 | | 6 | 4 | 6 | 6 | | 6 | 6 | 6 | 3 | | 7 | 7 | 7 | 5 | | 4 | 7 | 7 | 7 | | 2 | 1 | 1 | 2 | | 6 | 4 | 6 | 6 | |
| **23** | 2 | 1 | 2 | 4 | | 7 | 6 | 7 | 7 | | 7 | 7 | 7 | 6 | | 4 | 7 | 7 | 7 | | 6 | 7 | 7 | 7 | | 7 | 7 | 7 | 6 | | 6 | 7 | 6 | 6 | | 7 | 7 | 7 | 7 | | 2 | 1 | 2 | 1 | | 2 | 4 | 2 | 3 | |
| Average | **3.8** | | | | **4.5** | | | | | **4** | | | | | **5.4** | | | | | **3.8** | | | | | **3** | | | | | **4.4** | | | | | **4.5** | | | | | **5.1** | | | | | **5.3** | | | | |  |

### Table S2. Score details of AGREE II (articles 11-21)

| Reviewer | A | B | C | D | A | B | C | D | A | B | C | D | A | B | C | D | A | B | C | D | A | B | C | D | A | B | C | D | A | B | C | D | A | B | C | D | A | B | C | D | A | B | C | D |
| --- | --- | --- | --- | --- | --- | --- | --- | --- | --- | --- | --- | --- | --- | --- | --- | --- | --- | --- | --- | --- | --- | --- | --- | --- | --- | --- | --- | --- | --- | --- | --- | --- | --- | --- | --- | --- | --- | --- | --- | --- | --- | --- | --- | --- |
| **Section/Item** | Article11 | | | | Article12 | | | | Article13 | | | | Article14 | | | | Article15 | | | | Article16 | | | | Article17 | | | | Article18 | | | | Article19 | | | | Article20 | | | | Article21 | | | |
| **Purpose and scope.** | | | | | | | | | | | | | | | | | | | | | | | | | | | | | | | | | | | | | | | | | | | | |
| **1** | 6 | 6 | 6 | 7 | 6 | 7 | 7 | 6 | 6 | 5 | 6 | 7 | 6 | 6 | 7 | 7 | 6 | 7 | 7 | 6 | 6 | 6 | 6 | 7 | 7 | 6 | 6 | 7 | 5 | 6 | 5 | 7 | 5 | 6 | 5 | 7 | 4 | 5 | 5 | 5 | 5 | 6 | 6 | 6 |
| **2** | 6 | 4 | 6 | 6 | 6 | 6 | 7 | 7 | 6 | 4 | 6 | 7 | 6 | 5 | 6 | 6 | 6 | 6 | 6 | 6 | 6 | 5 | 6 | 6 | 5 | 6 | 6 | 6 | 6 | 6 | 6 | 6 | 6 | 6 | 6 | 6 | 5 | 5 | 5 | 5 | 6 | 6 | 6 | 6 |
| **3** | 5 | 3 | 5 | 5 | 5 | 4 | 3 | 4 | 6 | 6 | 6 | 7 | 4 | 3 | 4 | 5 | 6 | 5 | 5 | 5 | 6 | 7 | 6 | 7 | 7 | 6 | 6 | 7 | 4 | 5 | 4 | 4 | 6 | 6 | 6 | 7 | 4 | 5 | 5 | 5 | 4 | 5 | 5 | 5 |
| **Stakeholder involvement.** | | | | | | | | | | | | | | | | | | | | | | | | | | | | | | | | | | | | | | | | | | | | |
| **4** | 6 | 6 | 6 | 6 | 5 | 6 | 6 | 6 | 6 | 4 | 6 | 5 | 5 | 6 | 6 | 5 | 3 | 2 | 2 | 2 | 6 | 6 | 6 | 6 | 6 | 6 | 6 | 6 | 5 | 6 | 5 | 6 | 6 | 6 | 6 | 7 | 5 | 5 | 5 | 5 | 4 | 5 | 5 | 5 |
| **5** | 6 | 6 | 6 | 6 | 2 | 2 | 1 | 1 | 5 | 2 | 5 | 1 | 2 | 1 | 1 | 2 | 2 | 1 | 1 | 1 | 2 | 4 | 2 | 2 | 1 | 2 | 2 | 1 | 1 | 2 | 1 | 1 | 1 | 2 | 1 | 1 | 1 | 2 | 2 | 1 | 1 | 2 | 1 | 1 |
| **6** | 6 | 4 | 6 | 7 | 4 | 3 | 4 | 3 | 6 | 6 | 6 | 7 | 4 | 3 | 4 | 5 | 4 | 5 | 5 | 6 | 6 | 6 | 6 | 6 | 7 | 6 | 6 | 6 | 4 | 5 | 4 | 5 | 6 | 5 | 6 | 7 | 4 | 5 | 5 | 5 | 4 | 6 | 6 | 6 |
| **Rigor of development.** | | | | | | | | | | | | | | | | | | | | | | | | | | | | | | | | | | | | | | | | | | | | |
| **7** | 7 | 6 | 7 | 7 | 1 | 1 | 1 | 1 | 3 | 2 | 3 | 3 | 2 | 3 | 3 | 2 | 1 | 1 | 2 | 1 | 6 | 6 | 6 | 6 | 1 | 3 | 2 | 1 | 1 | 2 | 1 | 1 | 1 | 2 | 1 | 1 | 1 | 2 | 1 | 1 | 1 | 2 | 1 | 1 |
| **8** | 6 | 5 | 6 | 7 | 1 | 1 | 1 | 1 | 4 | 2 | 2 | 2 | 2 | 1 | 2 | 1 | 2 | 1 | 1 | 1 | 4 | 5 | 4 | 4 | 2 | 3 | 3 | 2 | 2 | 2 | 2 | 1 | 3 | 2 | 3 | 2 | 6 | 6 | 6 | 5 | 4 | 5 | 4 | 3 |
| **9** | 6 | 4 | 6 | 6 | 1 | 1 | 1 | 1 | 6 | 3 | 4 | 4 | 2 | 1 | 2 | 1 | 2 | 1 | 2 | 1 | 2 | 4 | 6 | 3 | 4 | 5 | 4 | 3 | 3 | 2 | 3 | 2 | 4 | 3 | 4 | 2 | 6 | 6 | 6 | 5 | 5 | 6 | 5 | 5 |
| **10** | 6 | 6 | 6 | 6 | 1 | 1 | 1 | 1 | 5 | 4 | 4 | 4 | 3 | 3 | 3 | 3 | 4 | 4 | 5 | 4 | 4 | 6 | 4 | 6 | 6 | 6 | 6 | 5 | 4 | 3 | 4 | 4 | 5 | 6 | 5 | 5 | 7 | 7 | 7 | 6 | 6 | 6 | 6 | 5 |
| **11** | 3 | 4 | 3 | 3 | 4 | 2 | 2 | 2 | 3 | 2 | 2 | 2 | 3 | 4 | 3 | 3 | 3 | 2 | 3 | 3 | 5 | 5 | 4 | 4 | 4 | 5 | 4 | 4 | 3 | 4 | 3 | 3 | 4 | 3 | 4 | 3 | 6 | 6 | 6 | 5 | 6 | 6 | 6 | 5 |
| **12** | 6 | 4 | 6 | 6 | 2 | 1 | 1 | 1 | 4 | 1 | 5 | 5 | 4 | 3 | 3 | 3 | 3 | 2 | 4 | 3 | 3 | 4 | 1 | 4 | 3 | 5 | 3 | 4 | 2 | 2 | 2 | 2 | 3 | 4 | 4 | 3 | 6 | 6 | 6 | 6 | 4 | 5 | 4 | 4 |
| **13** | 5 | 6 | 6 | 6 | 1 | 1 | 1 | 1 | 6 | 1 | 7 | 7 | 4 | 5 | 5 | 4 | 1 | 1 | 1 | 1 | 2 | 4 | 2 | 1 | 2 | 2 | 2 | 2 | 2 | 2 | 1 | 1 | 2 | 3 | 3 | 1 | 2 | 2 | 2 | 1 | 2 | 2 | 2 | 2 |
| **14** | 4 | 3 | 5 | 4 | 1 | 1 | 1 | 1 | 3 | 5 | 5 | 5 | 1 | 1 | 1 | 1 | 2 | 1 | 2 | 2 | 2 | 4 | 3 | 2 | 1 | 2 | 1 | 5 | 1 | 2 | 2 | 2 | 1 | 2 | 2 | 2 | 1 | 2 | 1 | 2 | 2 | 3 | 2 | 2 |
| **Clarity of presentation.** | | | | | | | | | | | | | | | | | | | | | | | | | | | | | | | | | | | | | | | | | | | | |
| **15** | 6 | 6 | 6 | 7 | 4 | 4 | 4 | 4 | 6 | 2 | 6 | 6 | 5 | 6 | 6 | 5 | 6 | 6 | 7 | 6 | 4 | 5 | 5 | 5 | 7 | 7 | 7 | 7 | 3 | 4 | 4 | 4 | 4 | 5 | 5 | 5 | 6 | 6 | 6 | 6 | 5 | 6 | 5 | 5 |
| **16** | 5 | 4 | 5 | 4 | 3 | 4 | 4 | 4 | 6 | 2 | 6 | 4 | 2 | 3 | 3 | 4 | 5 | 5 | 6 | 5 | 4 | 5 | 5 | 5 | 6 | 6 | 6 | 5 | 3 | 3 | 3 | 3 | 3 | 4 | 4 | 3 | 6 | 6 | 5 | 5 | 5 | 6 | 5 | 5 |
| **17** | 6 | 6 | 6 | 7 | 3 | 3 | 4 | 5 | 6 | 2 | 6 | 5 | 3 | 3 | 3 | 3 | 5 | 5 | 7 | 5 | 4 | 5 | 5 | 5 | 7 | 7 | 7 | 5 | 2 | 1 | 4 | 4 | 2 | 2 | 2 | 5 | 7 | 7 | 7 | 7 | 5 | 6 | 6 | 6 |
| **Applicability.** | | | | | | | | | | | | | | | | | | | | | | | | | | | | | | | | | | | | | | | | | | | | |
| **18** | 6 | 6 | 6 | 6 | 2 | 3 | 3 | 2 | 4 | 3 | 4 | 4 | 2 | 1 | 1 | 2 | 5 | 6 | 6 | 5 | 4 | 4 | 4 | 6 | 6 | 6 | 6 | 2 | 3 | 4 | 4 | 4 | 5 | 6 | 5 | 5 | 6 | 6 | 5 | 5 | 6 | 6 | 5 | 5 |
| **19** | 6 | 5 | 6 | 5 | 2 | 2 | 3 | 2 | 6 | 4 | 6 | 6 | 2 | 2 | 2 | 2 | 5 | 5 | 4 | 5 | 3 | 5 | 3 | 5 | 6 | 6 | 6 | 2 | 4 | 4 | 3 | 3 | 5 | 6 | 6 | 6 | 6 | 6 | 2 | 2 | 5 | 6 | 2 | 2 |
| **20** | 6 | 4 | 6 | 6 | 2 | 2 | 3 | 3 | 6 | 4 | 6 | 5 | 1 | 1 | 1 | 1 | 5 | 4 | 5 | 5 | 4 | 4 | 4 | 3 | 5 | 6 | 5 | 6 | 4 | 4 | 4 | 4 | 5 | 5 | 5 | 5 | 6 | 6 | 6 | 6 | 5 | 6 | 5 | 5 |
| **21** | 5 | 3 | 5 | 5 | 2 | 2 | 3 | 2 | 3 | 5 | 5 | 3 | 5 | 5 | 5 | 5 | 5 | 5 | 6 | 5 | 3 | 3 | 3 | 2 | **3** | **5** | **5** | **6** | 2 | 1 | 2 | 2 | **2** | **3** | **2** | **2** | 5 | 6 | 6 | 6 | 4 | 5 | 4 | 4 |
| **Independence.** | | | | | | | | | | | | | | | | | | | | | | | | | | | | | | | | | | | | | | | | | | | | |
| **22** | 6 | 7 | 6 | 6 | 1 | 2 | 1 | 1 | 6 | 6 | 6 | 7 | 5 | 6 | 6 | 5 | 6 | 7 | 7 | 7 | 6 | 7 | 6 | 7 | 2 | 2 | 1 | 1 | 7 | 6 | 5 | 5 | 7 | 7 | 5 | 5 | 6 | 7 | 6 | 6 | 6 | 7 | 6 | 6 |
| **23** | 5 | 7 | 5 | 5 | 5 | 6 | 6 | 5 | 6 | 6 | 6 | 7 | 6 | 6 | 6 | 6 | 6 | 6 | 7 | 6 | 6 | 6 | 6 | 6 | 2 | 2 | 1 | 1 | 7 | 7 | 6 | 6 | 7 | 7 | 7 | 7 | 7 | 7 | 7 | 7 | 6 | 6 | 6 | 6 |
| Average | **5.5** | | | | **2.8** | | | | **4.7** | | | | **3.5** | | | | **4.1** | | | | **4.6** | | | | **4.4** | | | | **3.5** | | | | **4.2** | | | | **4.9** | | | | **4.6** | | | |

### Table S3. Score details of AGREE II (articles 22-31)

| Reviewer | A | B | C | D | A | B | C | D | A | B | C | D | A | B | C | D | A | B | C | D | A | B | C | D | A | B | C | D | A | B | C | D | A | B | C | D | A | B | C | D |
| --- | --- | --- | --- | --- | --- | --- | --- | --- | --- | --- | --- | --- | --- | --- | --- | --- | --- | --- | --- | --- | --- | --- | --- | --- | --- | --- | --- | --- | --- | --- | --- | --- | --- | --- | --- | --- | --- | --- | --- | --- |
| **Section/Item** | Article22 | | | | Article23 | | | | Article24 | | | | Article25 | | | | Article26 | | | | Article27 | | | | Article28 | | | | Article29 | | | | Article30 | | | | Article31 | | | |
| **Purpose and scope.** | | | | | | | | | | | | | | | | | | | | | | | | | | | | | | | | | | | | | | | | |
| **1** | 6 | 6 | 6 | 6 | 5 | 6 | 5 | 6 | 7 | 7 | 7 | 7 | 7 | 7 | 7 | 7 | 6 | 7 | 6 | 6 | 6 | 6 | 7 | 6 | 6 | 7 | 6 | 6 | 6 | 7 | 7 | 6 | 6 | 7 | 6 | 6 | 6 | 6 | 7 | 7 |
| **2** | 6 | 6 | 6 | 6 | 6 | 6 | 6 | 6 | 6 | 6 | 6 | 7 | 7 | 6 | 6 | 6 | 7 | 7 | 7 | 7 | 7 | 7 | 6 | 7 | 6 | 6 | 5 | 6 | 6 | 6 | 6 | 6 | 6 | 6 | 7 | 6 | 6 | 7 | 6 | 6 |
| **3** | 5 | 6 | 5 | 5 | 4 | 5 | 4 | 6 | 6 | 7 | 6 | 6 | 2 | 2 | 2 | 2 | 2 | 1 | 1 | 2 | 2 | 2 | 2 | 2 | 2 | 2 | 1 | 2 | 5 | 3 | 4 | 5 | 5 | 5 | 5 | 5 | 2 | 2 | 1 | 1 |
| **Stakeholder involvement.** | | | | | | | | | | | | | | | | | | | | | | | | | | | | | | | | | | | | | | | | |
| **4** | 5 | 6 | 5 | 6 | 4 | 5 | 4 | 5 | 5 | 5 | 5 | 5 | 3 | 2 | 2 | 2 | 3 | 4 | 3 | 3 | 3 | 3 | 4 | 3 | 6 | 6 | 5 | 6 | 1 | 1 | 1 | 1 | 5 | 6 | 6 | 5 | 2 | 1 | 1 | 1 |
| **5** | 1 | 2 | 1 | 1 | 1 | 2 | 1 | 1 | 2 | 2 | 3 | 2 | 1 | 1 | 1 | 1 | 1 | 2 | 1 | 1 | 1 | 1 | 1 | 1 | 1 | 1 | 1 | 1 | 1 | 1 | 1 | 1 | 2 | 1 | 1 | 1 | 1 | 1 | 2 | 2 |
| **6** | 5 | 6 | 5 | 6 | 4 | 6 | 4 | 6 | 6 | 6 | 7 | 6 | 3 | 1 | 2 | 3 | 3 | 3 | 4 | 3 | 3 | 2 | 2 | 3 | 1 | 1 | 1 | 1 | 4 | 3 | 2 | 3 | 4 | 4 | 3 | 4 | 1 | 1 | 1 | 1 |
| **Rigor of development.** | | | | | | | | | | | | | | | | | | | | | | | | | | | | | | | | | | | | | | | | |
| **7** | 1 | 2 | 1 | 1 | 1 | 3 | 1 | 1 | 1 | 1 | 1 | 1 | 2 | 1 | 1 | 2 | 2 | 1 | 2 | 2 | 2 | 1 | 1 | 1 | 1 | 1 | 1 | 1 | 2 | 1 | 1 | 1 | 6 | 6 | 7 | 6 | 1 | 1 | 1 | 1 |
| **8** | 4 | 5 | 4 | 4 | 4 | 4 | 1 | 1 | 1 | 1 | 1 | 1 | 2 | 2 | 2 | 1 | 2 | 1 | 2 | 2 | 2 | 1 | 1 | 2 | 2 | 2 | 2 | 2 | 1 | 2 | 1 | 2 | 5 | 5 | 6 | 5 | 1 | 1 | 1 | 1 |
| **9** | 4 | 5 | 4 | 5 | 3 | 2 | 2 | 2 | 2 | 1 | 1 | 2 | 2 | 2 | 2 | 1 | 2 | 2 | 1 | 1 | 2 | 2 | 2 | 1 | 2 | 1 | 2 | 2 | 2 | 2 | 2 | 2 | 3 | 2 | 3 | 2 | 1 | 1 | 1 | 1 |
| **10** | 4 | 4 | 4 | 4 | 5 | 5 | 3 | 3 | 1 | 1 | 1 | 1 | 3 | 3 | 3 | 1 | 3 | 3 | 2 | 3 | 3 | 2 | 3 | 3 | 3 | 3 | 1 | 3 | 4 | 3 | 4 | 4 | 5 | 4 | 5 | 5 | 1 | 1 | 1 | 1 |
| **11** | 3 | 4 | 3 | 3 | 5 | 6 | 5 | 5 | 5 | 5 | 5 | 5 | 2 | 2 | 2 | 2 | 2 | 2 | 1 | 2 | 2 | 2 | 2 | 2 | 3 | 3 | 3 | 1 | 3 | 3 | 3 | 3 | 3 | 2 | 2 | 2 | 1 | 1 | 1 | 1 |
| **12** | 4 | 5 | 4 | 3 | 4 | 4 | 3 | 3 | 2 | 2 | 2 | 2 | 3 | 3 | 2 | 3 | 3 | 3 | 3 | 3 | 3 | 3 | 2 | 3 | 2 | 2 | 2 | 2 | 2 | 1 | 2 | 2 | 2 | 1 | 3 | 2 | 1 | 1 | 1 | 1 |
| **13** | 2 | 3 | 2 | 1 | 2 | 3 | 2 | 2 | 1 | 1 | 1 | 1 | 1 | 1 | 1 | 1 | 1 | 1 | 1 | 1 | 1 | 1 | 1 | 1 | 1 | 1 | 1 | 1 | 1 | 1 | 1 | 1 | 1 | 1 | 1 | 1 | 1 | 1 | 1 | 1 |
| **14** | 1 | 2 | 1 | 1 | 2 | 3 | 2 | 2 | 1 | 1 | 1 | 1 | 1 | 1 | 1 | 1 | 1 | 1 | 1 | 1 | 1 | 1 | 1 | 1 | 1 | 1 | 1 | 1 | 1 | 1 | 1 | 1 | 3 | 2 | 1 | 1 | 1 | 1 | 1 | 1 |
| **Clarity of presentation** | | | | | | | | | | | | | | | | | | | | | | | | | | | | | | | | | | | | | | | | |
| **15** | 3 | 4 | 4 | 3 | 4 | 5 | 4 | 4 | 6 | 6 | 6 | 6 | 5 | 6 | 6 | 5 | 5 | 6 | 6 | 6 | 5 | 6 | 5 | 5 | 5 | 6 | 6 | 5 | 5 | 6 | 5 | 6 | 5 | 5 | 6 | 5 | 6 | 6 | 6 | 6 |
| **16** | 4 | 3 | 3 | 3 | 4 | 5 | 4 | **4** | 6 | 6 | 6 | 6 | 5 | 5 | 5 | 5 | 5 | 6 | 6 | 5 | 5 | 5 | 6 | 5 | 5 | 5 | 5 | 6 | 4 | 5 | 5 | 4 | 5 | 5 | 6 | 5 | 5 | 6 | 6 | 6 |
| **17** | 3 | 4 | 4 | 5 | **3** | **2** | **3** | **4** | **6** | **6** | **6** | **6** | 5 | 5 | 5 | 5 | 5 | 5 | 6 | 5 | 5 | 6 | 5 | 5 | 5 | 5 | 5 | 5 | 4 | 5 | 5 | 4 | 5 | 5 | 6 | 5 | 6 | 6 | 6 | 6 |
| **Applicability.** | | | | | | | | | | | | | | | | | | | | | | | | | | | | | | | | | | | | | | | | |
| **18** | 4 | 5 | 5 | 5 | 5 | 6 | 5 | 5 | 5 | 5 | 5 | 5 | 2 | 3 | 3 | 2 | 4 | 4 | 5 | 4 | 2 | 2 | 2 | 2 | 5 | 5 | 4 | 5 | 2 | 3 | 2 | 1 | 3 | 4 | 2 | 3 | 1 | 2 | 2 | 2 |
| **19** | 4 | 4 | 4 | 3 | 4 | 4 | 4 | 4 | 2 | 2 | 3 | 2 | 2 | 3 | 2 | 3 | 2 | 2 | 1 | 2 | 2 | 2 | 2 | 2 | 4 | 3 | 4 | 3 | 2 | 2 | 1 | 2 | 5 | 5 | 3 | 5 | 1 | 1 | 1 | 1 |
| **20** | 3 | 4 | 4 | 4 | 4 | 3 | 4 | 3 | 4 | 4 | 4 | 4 | 2 | 2 | 2 | 2 | 2 | 2 | 2 | 2 | 2 | 2 | 2 | 2 | 4 | 4 | 3 | 4 | 4 | 5 | 4 | 4 | 5 | 5 | 6 | 5 | 1 | 2 | 3 | 1 |
| **21** | 3 | 5 | 5 | 2 | 2 | 3 | 2 | 2 | 6 | 6 | 6 | 6 | ~~5~~ | ~~3~~ | ~~4~~ | ~~5~~ | ~~5~~ | ~~5~~ | ~~6~~ | ~~5~~ | ~~5~~ | ~~4~~ | ~~5~~ | ~~5~~ | 5 | 5 | 6 | 5 | 4 | 5 | 5 | 4 | 5 | 5 | 5 | 5 | 5 | 5 | 5 | 5 |
| **Independence.** | | | | | | | | | | | | | | | | | | | | | | | | | | | | | | | | | | | | | | | | |
| **22** | 6 | 7 | 6 | 6 | 6 | 6 | 6 | 6 | 2 | 2 | 2 | 2 | 1 | 1 | 1 | 1 | 5 | 5 | 6 | 5 | 5 | 5 | 6 | 5 | 4 | 5 | 4 | 4 | 1 | 1 | 1 | 1 | 6 | 6 | 7 | 6 | 1 | 1 | 1 | 1 |
| **23** | 7 | 7 | 7 | 6 | 6 | 6 | 6 | 6 | 6 | 6 | 7 | 6 | 6 | 7 | 7 | 6 | 6 | 6 | 5 | 6 | 6 | 6 | 6 | 6 | 6 | 6 | 6 | 6 | 1 | 2 | 1 | 1 | 6 | 7 | 6 | 6 | 1 | 1 | 1 | 1 |
| Average | **4.1** | | | | **3.9** | | | | **3.9** | | | | **3** | | | | **3.4** | | | | **3.2** | | | | **3.4** | | | | **2.9** | | | | **4.3** | | | | **2.4** | | | |

### Table S4. Score details of AGREE II (article 32-36)

| Reviewer | A | B | C | D | A | B | C | D | A | B | C | D | A | B | C | D | A | B | C | D |
| --- | --- | --- | --- | --- | --- | --- | --- | --- | --- | --- | --- | --- | --- | --- | --- | --- | --- | --- | --- | --- |
| **Section/Item** | Article32 | | | | Article33 | | | | Article34 | | | | Article35 | | | | Article36 | | | |
| **Purpose and scope.** | | | | | | | | | | | | | | | | | | | | |
| **1** | 6 | 6 | 6 | 7 | 5 | 6 | 7 | 6 | 6 | 6 | 7 | 6 | 6 | 7 | 7 | 7 | 6 | 7 | 6 | 7 |
| **2** | 6 | 6 | 5 | 6 | 5 | 6 | 6 | 5 | 6 | 6 | 7 | 6 | 6 | 7 | 6 | 6 | 6 | 7 | 6 | 6 |
| **3** | 5 | 4 | 5 | 4 | 2 | 1 | 1 | 2 | 4 | 4 | 3 | 4 | 6 | 6 | 7 | 6 | 2 | 1 | 2 | 2 |
| **Stakeholder involvement.** | | | | | | | | | | | | | | | | | | | | |
| **4** | 7 | 6 | 5 | 6 | 1 | 1 | 2 | 2 | 5 | 6 | 6 | 5 | 7 | 7 | 7 | 7 | 2 | 1 | 1 | 1 |
| **5** | 3 | 1 | 2 | 2 | 1 | 1 | 1 | 2 | 2 | 1 | 1 | 1 | 5 | 6 | 6 | 6 | 1 | 1 | 1 | 1 |
| **6** | 4 | 2 | 3 | 3 | 1 | 1 | 1 | 1 | 4 | 3 | 3 | 3 | 6 | 5 | 5 | 6 | 2 | 2 | 2 | 2 |
| **Rigor of development.** | | | | | | | | | | | | | | | | | | | | |
| **7** | 6 | 7 | 6 | 6 | 1 | 2 | 1 | 1 | 2 | 2 | 2 | 2 | 2 | 1 | 3 | 2 | 1 | 1 | 1 | 1 |
| **8** | 3 | 2 | 2 | 2 | 3 | 2 | 2 | 3 | 4 | 3 | 2 | 1 | 3 | 2 | 3 | 3 | 1 | 1 | 1 | 1 |
| **9** | 2 | 1 | 1 | 2 | 1 | 1 | 1 | 1 | 2 | 1 | 1 | 2 | 3 | 3 | 3 | 3 | 1 | 1 | 1 | 1 |
| **10** | 5 | 4 | 4 | 4 | 1 | 1 | 1 | 1 | 2 | 2 | 2 | 2 | 4 | 5 | 6 | 4 | 1 | 1 | 2 | 1 |
| **11** | 5 | 5 | 5 | 5 | 3 | 2 | 2 | 3 | 4 | 4 | 4 | 4 | 7 | 7 | 7 | 7 | 1 | 2 | 1 | 1 |
| **12** | 3 | 2 | 2 | 3 | 2 | 1 | 1 | 2 | 1 | 1 | 1 | 1 | 4 | 3 | 3 | 4 | 1 | 1 | 1 | 1 |
| **13** | 1 | 1 | 1 | 1 | 1 | 1 | 1 | 1 | 1 | 1 | 1 | 1 | 6 | 7 | 7 | 6 | 1 | 1 | 1 | 1 |
| **14** | 1 | 1 | 1 | 1 | 1 | 1 | 1 | 1 | 4 | 5 | 4 | 4 | 7 | 6 | 6 | 7 | 1 | 1 | 1 | 1 |
| **Clarity of presentation** | | | | | | | | | | | | | | | | | | | | |
| **15** | 6 | 6 | 6 | 6 | 5 | 5 | 6 | 6 | 5 | 6 | 6 | 6 | 7 | 7 | 7 | 7 | 5 | 5 | 5 | 5 |
| **16** | 5 | 5 | 6 | 5 | 5 | 6 | 5 | 5 | 5 | 5 | 5 | 5 | 7 | 7 | 7 | 7 | 5 | 5 | 5 | 5 |
| **17** | 6 | 5 | 4 | 3 | 5 | 6 | 5 | 5 | 5 | 6 | 6 | 6 | 7 | 7 | 7 | 7 | 5 | 5 | 5 | 5 |
| **Applicability.** | | | | | | | | | | | | | | | | | | | | |
| **18** | 5 | 4 | 4 | 5 | 4 | 5 | 3 | 4 | 5 | 5 | 6 | 6 | 6 | 6 | 7 | 7 | 2 | 1 | 2 | 2 |
| **19** | 5 | 5 | 4 | 4 | 4 | 5 | 4 | 5 | 5 | 4 | 5 | 5 | 6 | 6 | 6 | 6 | 2 | 1 | 2 | 2 |
| **20** | 5 | 4 | 5 | 5 | 5 | 4 | 5 | 6 | 4 | 3 | 3 | 3 | 6 | 6 | 6 | 6 | 2 | 1 | 2 | 2 |
| **21** | 5 | 4 | 4 | 4 | 5 | 3 | 6 | 5 | 5 | 5 | 5 | 5 | 6 | 6 | 6 | 6 | 2 | 1 | 2 | 2 |
| **Independence.** | | | | | | | | | | | | | | | | | | | | |
| **22** | 4 | 5 | 4 | 6 | 5 | 6 | 7 | 5 | 2 | 1 | 1 | 1 | 1 | 2 | 2 | 2 | 1 | 1 | 1 | 1 |
| **23** | 2 | 1 | 1 | 1 | 6 | 6 | 6 | 6 | 6 | 7 | 7 | 7 | 1 | 2 | 2 | 1 | 1 | 1 | 1 | 1 |
| Average | **4** | | | | **3.2** | | | | **3.8** | | | | **5.3** | | | | **2.2** | | | |

### Table S5. AGREE II percentage quality assessment of a scaled domain

| No. | **Purpose and scope.** | **Stakeholder involvement.** | **Rigor of development.** | **Clarity of presentation.** | **Applicability.** | **Independence.** | **Cronbach's Alpha** |
| --- | --- | --- | --- | --- | --- | --- | --- |
| Article1 | 66.7% | 29.2% | 34.4% | 68.1% | 56.3% | 50.0% | **0.90** |
| Article 2 | 58.3% | 38.9% | 51.6% | 19.4% | 51.0% | 95.8% | **0.90** |
| Article 3 | 76.4% | 80.6% | 72.9% | 77.8% | 68.8% | 52.1% | **0.89** |
| Article 4 | 73.6% | 81.9% | 72.4% | 77.8% | 72.9% | 81.3% | **0.85** |
| Article 5 | 75.0% | 81.9% | 82.8% | 68.1% | 60.4% | 60.4% | **0.82** |
| Article 6 | 83.3% | 65.3% | 44.8% | 62.5% | 60.4% | 87.5% | **0.81** |
| Article 7 | 72.2% | 45.8% | 21.9% | 59.7% | 53.1% | 85.4% | **0.94** |
| Article 8 | 86.1% | 63.9% | 49.0% | 62.5% | 45.8% | 87.5% | **0.92** |
| Article 9 | 52.8% | 40.3% | 18.8% | 6.9% | 40.6% | 83.3% | **0.97** |
| Article 10 | 72.2% | 45.8% | 20.3% | 36.1% | 37.5% | 85.4% | **0.97** |
| Article 11 | 79.2% | 45.8% | 40.1% | 81.9% | 47.9% | 89.6% | **0.98** |
| Article 12 | 83.3% | 58.3% | 31.8% | 44.4% | 59.4% | 91.7% | **0.96** |
| Article 13 | 87.5% | 59.7% | 38.0% | 90.3% | 67.7% | 8.3% | **0.94** |
| Article 14 | 63.9% | 45.8% | 55.2% | 86.1% | 71.9% | 93.8% | **0.97** |
| Article 15 | 80.6% | 44.4% | 46.9% | 75.0% | 45.8% | 93.8% | **0.97** |
| Article 16 | 75.0% | 47.2% | 46.9% | 73.6% | 61.5% | 85.4% | **0.97** |
| Article 17 | 79.2% | 51.4% | 34.4% | 43.1% | 50.0% | 91.7% | **0.97** |
| Article 18 | 80.6% | 47.2% | 41.7% | 65.3% | 58.3% | 93.8% | **0.93** |
| Article 19 | 73.6% | 43.1% | 32.3% | 47.2% | 45.8% | 83.3% | **0.95** |
| Article 20 | 90.3% | 91.7% | 63.0% | 88.9% | 57.3% | 8.3% | **0.98** |
| Article 21 | 91.7% | 58.3% | 11.5% | 83.3% | 55.2% | 52.1% | **1.00** |
| Article 22 | 91.7% | 61.1% | 49.5% | 87.5% | 75.0% | 50.0% | **0.98** |
| Article 23 | 76.4% | 43.1% | 28.6% | 87.5% | 71.9% | 85.4% | **0.99** |
| Article 24 | 93.1% | 27.8% | 31.8% | 80.6% | 52.1% | 18.8% | **0.98** |
| Article 25 | 72.2% | 20.8% | 24.5% | 88.9% | 76.0% | 97.9% | **0.98** |
| Article 26 | 97.2% | 58.3% | 53.6% | 87.5% | 74.0% | 104.2% | **0.97** |
| Article 27 | 94.4% | 59.7% | 20.3% | 63.9% | 39.6% | 56.3% | **0.98** |
| Article 28 | 90.3% | 55.6% | 38.0% | 91.7% | 77.1% | 66.7% | **0.98** |
| Article 29 | 90.3% | 61.1% | 42.7% | 63.9% | 39.6% | 95.8% | **0.98** |
| Article 30 | 98.6% | 47.2% | 34.9% | 94.4% | 84.4% | 108.3% | **0.98** |
| Article 31 | 79.2% | 20.8% | 16.7% | 98.6% | 39.6% | 16.7% | **0.99** |
| Article 32 | 106.9% | 101.4% | 75.0% | 116.7% | 102.1% | 27.1% | **0.99** |
| Article 33 | 80.6% | 23.6% | 17.7% | 83.3% | 29.2% | 16.7% | **0.99** |
| Article 34 | 84.7% | 30.6% | 29.7% | 86.1% | 46.9% | 62.5% | **0.98** |
| Article 35 | 81.9% | 43.1% | 30.2% | 91.7% | 55.2% | 91.7% | **0.99** |
| Article 36 | 83.3% | 37.5% | 29.2% | 87.5% | 44.8% | 93.8% | **0.99** |
